# Chinese Multidisciplinary Expert Consensus on Orphan/Anticopper Drugs and Other Non-drug Management of Hepatolenticular Degeneration

**DOI:** 10.2174/011570159X349587250311072553

**Published:** 2025-04-07

**Authors:** Ren-Min Yang, Tao Feng, Wei Cai, Xu-En Yu, Gang Wang, Yong-Zhu Han, Cun-Xiu Fan, Qiang Xia, Hai-Bo Chen, Xiao-Ping Wang

**Affiliations:** 1 Department of Neurology, Institute of Neurology, Anhui University of Chinese Medicine, Hefei, China;; 2 Department of Neurology, Tiantan Hospital Affiliated to Capital Medical University, Beijing, China;; 3 Division of Surgery Pediatrics, Shanghai Institute of Pediatrics, Shanghai, China;; 4 Department of Neurology, Ruijin Hospital Affiliated to Shanghai Jiao Tong University School of Medicine, Ruijin, China;; 5 Division of Basic Medicine, Shanghai International Academician Station (Jiading), Shanghai, China;; 6 Department of Neurology, Liver Transplantation Center, Renji Hospital, Affiliated to Shanghai Jiao Tong University School of Medicine, Shanghai, China;; 7 Department of Neurology, Institute of Geriatric Medicine, Beijing Hospital & National Center of Gerontology, Beijing, China;; 8 Jiading Branch, Shanghai General Hospital, Shanghai Jiao Tong University School of Medicine, Shanghai, China

**Keywords:** Multidisciplinary expert, consensus, guideline, Wilson's disease, anticopper drugs, hepatolenticular degeneration

## Abstract

**Background:**

This study aims to guide the diagnosis and treatment of hepatolenticular degeneration (also named Wilson's disease, WD) and aid multidisciplinary clinicians in making reasonable and personalized treatment regimens.

**Objectives:**

The authors aim to establish a systemic structure for Chinese Multidisciplinary Expert Consensus on Diagnosis and Treatment of Hepatolenticular Degeneration.

**Methods:**

We collaborated with experts from relevant branches of the Chinese Medical Association and multiple disciplines, along with statistical experts, to formulate this consensus. It is based on advancements in basic and clinical research on Wilson's disease, both domestically and internationally.

**Results:**

It mainly consists of clinical manifestations, diagnosis, differential diagnosis, management, and prognosis in the context of Multi-Department treatment (MDT) in China.

**Conclusion:**

This Chinese consensus incorporates four decades of institutional experience with thousands of Chinese Wilson’s disease (WD) inpatients, as well as decades of international inpatient cases from East to West. It is hoped that this consensus will garner broader attention from clinicians worldwide.

## 1 INTRODUCTION

Hepatolenticular degeneration, also known as Wilson's disease (WD), was first described in 1912 by Kinnear Wilson, a famous British neurologist from the Institute of Neurology Queen Square, University College London, in his doctoral dissertation. WD is caused by mutations in the ATPase copper transporting beta (ATP7B) gene located on chromosome 13q. These mutations lead to defective biliary copper excretion, resulting in excessive copper accumulation in multiple organs, such as the nervous system and digestive system, thus causing movement disorders, liver disease, psychiatric symptoms, and damage to the eyes, kidneys, and blood system, *etc*. The clinical manifestations of WD are complex, often leading to misdiagnosis and missed diagnosis. A comprehensive assessment and treatment by the multidisciplinary departments of physicians is required. Genetic testing, histopathologic examination, and specialized imaging techniques are also needed to confirm the diagnosis and guide treatment. Although Wilson’s disease (WD) is often considered a pediatric or young adult disease [1], it can affect individuals of all ages, with onset even reported at 70 years old. Aging patients also require regular management and long-term follow-up.

The American Association for the Study of Liver Diseases (AASLD) published (2023 wonderful Guidance worldly up to date) the latest version of clinical practice Guidance for the diagnosis and management of Wilson disease in September 2022 [1]. The British Association for the Study of the Liver released guidelines for the investigation and management of Wilson's disease in June 2022 [2]. In China, the Neurology Branch of the Chinese Medical Association published the Chinese guidelines for the diagnosis and treatment of hepatolenticular degeneration in 2021, and the Hepatology Branch of the Chinese Medical Association published the Guidelines for the diagnosis and treatment of hepatolenticular degeneration in 2022. In recent years, with the progress of research on WD and the accumulation of experience in diagnosis and treatment, creating a multidisciplinary consensus/guideline on the diagnosis and treatment of WD has become particularly important. We invited experts from various fields, including neurology, pediatrics, hepatology surgery, gastroenterology, pathology, and clinical laboratory, to formulate multidisciplinary guidelines for the diagnosis and treatment of WD. We also received help and guidance from Prof. Michael Schilsky, the Chair of the International Hepatolenticular degeneration Forum, and other experts overseas.

As a relatively rare disorder, WD often lacks comprehensive data. Therefore, this consensus, rather than a formal guideline, is not a mandatory standard. Instead, it aims to provide guidance on the diagnosis and treatment of WD as much as possible, though it cannot fully cover or address all potential issues. We hope that further international and national multi-center randomized controlled trials will help refine and enhance the next multidisciplinary consensus or ultimately lead to the development of a formal guideline. When facing patients with suspected WD, clinicians should follow the basic principles of this consensus, thoroughly understand patients' conditions, and formulate comprehensive, reasonable, personalized diagnosis and treatment regimens in partnership with the multidisciplinary team based on their clinical practice experience.

According to the Grading of Recommendations, Assessment, Development, and Evaluation (GRADE) system, the evidence levels of this guideline are classified into A, B, and C, and the grades of recommendation are classified into 1 and 2 (Table **1**), and the professional direction from International Practice Guide Registration and Transparency Platform (No. PREPARE-2024CN115) http://www.guidelines-registry.cn/.

## 2 EPIDEMIOLOGY

WD can manifest at any age, but it occurs more commonly in children and adolescents. The usual age of onset is 4 to 40 years. The symptoms of WD can also appear in individuals below the age of 3 years and over the age of 80 years. The prevalence of WD is almost the same for both males and females. The neurological symptoms of WD are relatively more common in males, who tend to have an earlier disease onset and diagnosis, often presenting with various movement disorders. In contrast, the hepatic symptoms occur more frequently in females. The latest estimate indicates that there are more than 15,000 hospitalizations for WD among adult patients in the USA (not concluded non-adult cases), with an estimated prevalence of 1 per 8300 to 11,000 individuals. A study from the UK [3] showed that the genetic prevalence of WD is approximately 1:7026-1:20,000. Based on the frequency of ATP7B gene mutations in humans, it is speculated that the estimated prevalence of WD ranges from 1.21/10,000 to 1.96/10,000 in the UK. Chinese scholars Hu and Yang *et al*. conducted two surveys in three counties of Anhui province. A total of 153,370 individuals were examined, and 9 individuals with WD were identified, with a prevalence of 5.87/100,000. It is estimated that the prevalence of WD is about 1/10,000 in China, resulting in approximately 80,000-100,000 individuals with WD in mainland China.

## 3 PATHOGENESIS

The pathophysiology and clinical manifestations of WD are attributed to alterations in copper homeostasis. Copper is an important cofactor for several physiological enzymatic reactions in critical metabolic pathways and is thereby considered an essential trace element. Copper homeostasis is maintained through the gut, which is responsible for diet copper uptake and use. The liver removes excess copper in bile, which is then excreted into the stool. The hepatocytes utilize copper for metabolic demands, incorporate copper into nascent ceruloplasmin, and transport excess copper into the bile [1]. Most excess copper is excreted through the biliary pathway into feces. Only a small amount of copper is excreted through the kidneys. Therefore, impaired biliary copper excretion can mainly lead to copper retention in the liver [1].

WD is an autosomal recessive disorder due to the presence of mutations in the ATP7B gene localized on 13q14.3 [2, 3]. A large proportion of WD patients are compound heterozygotes for ATP7B mutations. A meta-analysis identified 782 ATP7B variants, with 216 being considered classified as “likely pathogenic variants” [4]. According to data from Cardiff University WD gene bank, more than 1000 ATP7B gene variants have been reported. Loss of functional ATP7B alters the excretion of copper from hepatocytes *via* the bile, as well as the biosynthetic incorporation of copper into ceruloplasmin. Ceruloplasmin is a serum ferroxidase that is responsible for 90% of copper transport from the liver. Loss of functional ATP7B also reduces hepatocellular copper incorporation into ceruloplasmin. The resulting ceruloplasmin has a short circulating half-life, leading to low steady-state levels of circulating ceruloplasmin [1, 5]. Excess copper caused by reduced biliary copper excretion leads to the production of free radicals that cause oxidation of vital proteins and lipids and alter methionine metabolism, thus resulting in DNA hypomethylation, epigenetic changes [1, 6, 7], as well as early damage affecting the mitochondria, nuclei, and peroxisomes. Since 95% of copper excretion is through the liver-bile, this excess copper first accumulates in the liver, causing varying degrees of liver damage, which eventually accumulates in other organs, notably the brain, kidney, and cornea [1, 8].

## 4 CLINICAL MANIFESTATIONS

Clinical manifestations of WD are diverse and vary depending on the organs involved and the degree of organ damage due to copper accumulation, which are predominantly hepatic and neurologic, as well as varied symptoms’ Kaleidoscope same as “Hysteria spectrum” nearly. WD patients with predominant liver involvement tend to have an earlier onset (after the first year of life) and are often diagnosed during kindergarten entrance physical examinations in mainland China. Neurological involvement often appears about 10 years after liver involvement, usually between 8 and 13 years of age. Additionally, WD patients can also have a variety of clinical manifestations, *e.g*., ophthalmological abnormalities, hemolysis, kidney damage, and bone and joint symptoms [1].

In WD patients, the progressive accumulation of copper in the liver begins in infancy when copper-containing solids are introduced into the diet. However, WD is rarely symptomatic before the age of 5 years [1, 9-11]. Neurological manifestations are rarely seen in children younger than 10 years [12], but mild impairments in memory and language development are frequently reported in the retrospective study. Kayser-Fleischer (K-F) rings are usually absent in asymptomatic children or those with mild liver disease but are present in children with neurological symptoms [1, 13]. Since the geriatric manifestation of WD is rare and data are mostly limited to case reports, the upper age limit for consideration of WD is generally 55 years. Previous molecular studies on the identification of mutations in the ATP7B gene confirmed that the oldest WD patients are in their early 70s [7, 14, 15]. A study of 46 WD patients who became symptomatic at > 40 years of age showed that among 27 patients with available liver biopsy specimens, 19 had cirrhosis, and 3 had no liver abnormalities. It has been found that the diagnostic features and frequency of gene mutations in patients with late-onset WD were not different from those in patients with early-onset WD [16]. All patients presenting with WD signs and symptoms should be further evaluated, regardless of age [1, 17, 18].

### 4.1 Neurological Manifestations

The typical neurological manifestations of WD are dysarthria, dystonia, dysphagia, excessive salivation, ataxia, gait abnormalities, rigidity, mask-like faces, *risus sardonicus* (being considered a characteristic facial manifestation of WD), insomnia, and epilepsy [1, 18]. However, this is only found in patients with advanced neurological manifestations [1]. WD patients who presented predominantly with neurological symptoms show elevated copper concentrations in the cerebrospinal fluid, which are 3 to 4 times higher than those in non-WD patients and WD patients without neurological manifestations. Most patients with neurological symptoms exhibit signs of liver involvement [1].

Behavioral or personality changes (incongruous behavior, irritability, aggression, and disinhibition), mood disorders (hypomania or depression), and anxiety are common in WD patients [18, 19]. Psychosis may also occur in WD and is usually accompanied by paranoia and hallucinations. The psychiatric manifestations of WD are often overlooked, particularly in the pediatric population, whose behavior or mood changes are considered a normal part of adolescence [20, 21]. Although screening WD patients for isolated psychiatric symptoms may have a low yield, psychiatrists should remain vigilant for hepatic (including coma prodrome) and/or neurological signs. If WD is suspected, prompt investigation and referral are crucial. [1].

Neurological manifestations (such as various types of tremor, mixed type of tremor, rigidity, dystonia, choreoathetosis, and dystonic tremor) were predominant in male patients (60%) than female patients (40%) [1, 22]. The mean age at onset of neurological symptoms was lower in male patients (27.1 years) than in female patients (29.4 years), and the mean age at diagnosis was also lower for male patients (29.8 years) compared to female patients (32 years). Central nervous system-related symptoms were more commonly seen in male patients (55%) than in female patients (45%). Neurological manifestations were more common in male patients (38.1%) than in female patients (29.8%) [1].

#### 4.1.1 Dystonia

Dystonia is a disorder characterized by sustained or intermittent muscle contractions that often cause repetitive movements and/or abnormal postures [22]. The prevalence of dystonia ranges between 11% and 65% in WD patients with neurological symptoms. It is often focal and segmental in the early stage and progresses gradually to generalized dystonia, which can seriously affect patients’ daily activities. The severity of dystonia can be only mild and usually worsens with disease progression, which is often accompanied by severe muscle spasms in the limbs during the late stage of the disease. Focal manifestations of dystonia include blepharospasm, cervical dystonia (torticollis), writer's cramp, and a dystonic facial expression with an exaggerated smile (*risus sardonicus*). Focal dystonia of the vocal cords, muscles of articulation, or swallowing muscles may cause the occurrence of dysphonia, dysarthria, or dysphagia and drooling. Dysarthria is the most common neurological symptom of WD, occurring in about 85% to 97% of patients with neurological manifestations. The types of dysarthria may vary among patients, including ataxic (irregular word spacing and volume), athetoid, hypophonic, or spastic dysarthria [1, 22].

#### 4.1.2 Tremor

Tremors can occur at rest or during active movement, including essential, intentional (kinetic), and postural tremors. Severe postural tremor is characterized by a “wing-beating” tremor, which can be quite difficult to differentiate from other neurological abnormalities such as hepatic encephalopathy. Flapping Tremor or asterixis, a specific kind of *negative* myoclonic represented often, had been considered only as “liver flap of hepatic encephalopathy” [1, 22]. Action tremor with hypotonia is not rare in WD.

#### 4.1.3 Limb Rigidity and Bradykinesia

Some WD patients develop limb rigidity, bradykinesia or hypokinesia, difficulty in writing, abnormally small handwriting, and slow walking speed. Those patients are easily misdiagnosed as Parkinson's disease. Young adults should be screened for WD when they have extrapyramidal symptoms, such as rigidity-tremor syndrome [1, 22].

#### 4.1.4 Cognitive Changes and Dementia

Some WD patients have relatively high IQs as top University professors, but most of these patients have cognitive impairment. Patients exhibiting movement disorders usually present with behavioral changes or cognitive decline from the outset. This decline occurs in about 25% of patients. Depending on the tests used and whether patients exhibit more hepatic or neurological symptoms, this figure could be as high as 40%.

Executive dysfunction is a frequent neurological manifestation of WD, which is subtle and easily overlooked during a brief consultation [23-25]. WD patients experiencing executive dysfunction may have difficulty with decision-making, problems with multitasking and flexible thinking or concentration, and exhibit impulsive behavior. Executive dysfunction can also affect other cognitive domains, including processing speed, memory, visual function, and social cognition. The most widely used tests in adults are the Mini-Mental State Examination and Montreal Cognitive Assessment, but these two tests are less sensitive in detecting mild cognitive impairment. More comprehensive screening tools, such as the Addenbrooke’s Cognitive Examination or formal neuropsychological tests, may be required [2, 26]. Wang *et al*. studied the Procedural Learning skill for movement disorders with implicit cognition decline.

#### 4.1.5 Psychiatric and Behavioral Abnormalities

Psychiatric and behavioral abnormalities are relatively common in WD patients and may even precede neurological and hepatic manifestations. However, these symptoms are easily overlooked. The diagnosis is often delayed until hepatic or neurological symptoms appear. Psychiatric symptoms vary widely, with affective disorders being the most common manifestations. Patients can also manifest personality changes, depression, cognitive changes, and anxiety. For adolescent patients, psychiatric and behavioral abnormalities may manifest as reduced learning capacity and mood swings, which can easily be confused with physiological, emotional changes, and personality changes during adolescence. Elderly patients can develop paranoid delusions, schizophrenia-like symptoms, depression and even suicide.

#### 4.1.6 Other Rare Neurological Symptoms

A few patients can develop other rare neurological symptoms, such as choreiform movements, athetosis, and striatal-like hands.

### 4.2 Hepatic Manifestations

WD can be considered a systemic or whole-body disease, requiring the attentive consideration of all physicians, even for cases with a low incidence. In terms of hepatic manifestations of WD, symptoms may be nonspecific, so an accurate method for assisting in the differential diagnosis of these symptoms is therefore required. WD can be clinically presented as asymptomatic or symptomatic [1]. Asymptomatic patients are usually detected during the kindergarten entrance and school physical examination or diagnosed accidentally during family screening. The findings include elevated aspartate aminotransferase (AST) and alanine aminotransferase (ALT) [1, 22, 27]. WD patients with symptomatic hepatitis can present with insidious onset of vague symptoms, including tiredness and loss of appetite, followed by jaundice. Other findings include acute hepatitis with a sharp elevation of liver enzymes, portal hypertension with or without variceal bleeding, incidental evidence of hepatomegaly, and eventually compensated and decompensated cirrhosis. Approximately 3-5% of WD patients present with acute liver failure (ALF) [1, 28], which is traditionally called “abdominal Wilsonian type”. Some patients have underlying fibrosis or cirrhosis that is not detected until acute clinical symptoms appear. ALF due to WD is usually characterized by jaundice, non-immune (Coombs-negative) intravascular hemolysis, coagulopathy, ascites, progressively hepatic coma, altered alkaline phosphatase (AlP): total bilirubin (TB) ratio (<1:4), altered AST: ALT ratio, and rapid progression of renal failure [29]. The mortality rate of ALF due to WD approaches 100% without liver transplantation [30].

Liver-related signs and symptoms are more common in female patients (56%) than in male patients (44%), and hepatic manifestations occur more frequently in females (56.5%) than in males (48.3%) [31].

#### 4.2.1 Asymptomatic Patients

Asymptomatic patients are defined as those who are only found to have elevated transaminases, hepatosplenomegaly, or fatty liver during routine physical examination or accidental finding of positive K-F rings in the cornea, without any clinical symptoms and with a confirmed diagnosis of WD being made after further examination. Asymptomatic patients can also be diagnosed during genetic screening for ATP7B gene mutation in first-degree relatives of a proband with WD. It is worth noting that the earliest clinical manifestation of WD in children or adolescents is mild to moderate fatty liver. It has been shown that 3% to 40% of patients have no obvious clinical symptoms.

#### 4.2.2 Acute Hepatitis

Acute hepatitis caused by WD is similar to that of other etiologies, which is characterized by elevated transaminases, jaundice, and abdominal discomfort. In some patients with mild acute hepatitis, symptoms can resolve spontaneously, whereas the condition of some patients with severe acute hepatitis may deteriorate rapidly, leading to liver failure.

#### 4.2.3 ALF

A small number of WD patients may develop hepatic decompensation in a short period of time and manifest as impaired hepatic synthetic function, jaundice, coagulopathy, and hepatic encephalopathy. The process of ALF occurs and progresses rapidly, with a high mortality rate, often requiring liver transplantation. Additionally, when ALF occurs, some WD patients have pre-existing advanced liver fibrosis or cirrhosis. Therefore, acute-on-chronic liver failure is actually considered.

ALF due to WD occurs mostly in children and adolescents which is more common in females. Patients with ALF due to WD often present with the following characteristics: Coombs-negative hemolytic anemia that is often accompanied by the features of acute intravascular hemolysis, such as fever and hemoglobinuria; coagulopathy that cannot be effectively treated by vitamin K therapy; and rapid progression to kidney failure with normal or decreased serum uric acid levels. It has been shown that a serum ALP: TB ratio < 4 and an AST: ALT ratio > 2.2 yield a sensitivity and specificity of 100% for the diagnosis of ALF induced by WD, but further validation is needed. It should be noted that ALF may not be accompanied by a decrease in serum ceruloplasmin, making early diagnosis difficult. In this case, even a Leipzig score of < 4 points could not exclude a diagnosis of WD. Other tests, such as genetic testing for ATP7B mutations, should be performed to confirm the diagnosis further. Derived from hematology's coagulation function index, the International Normalized Ratio (INR) serves as an objective measure of synthetic liver function alongside albumin. Model of End‐Stage Liver Disease (MELD) score may be calculated and followed. Patients with a MELD score >15 who are failing to improve after suitable anticopper periods should be considered for evaluation for liver transplantation.

#### 4.2.4 Chronic Hepatitis-mimic and Cirrhosis

WD patients with chronic hepatitis-mimic present with symptoms such as fatigue and loss of appetite. Physical examination reveals signs of chronic liver disease, such as *dim complexion* and *Palmar erythema*. Laboratory tests show abnormal liver function, such as elevated transaminases and bilirubin levels. With the development of the disease, chronic hepatitis gradually progresses to liver fibrosis, compensated or decompensated cirrhosis, resulting in the occurrence of complications such as splenomegaly, hypersplenism, ascites, esophageal-gastric fundal varices, and hepatic encephalopathy. It has been found that liver cirrhosis is present in about 35% to 45% of patients at the time of diagnosis of WD, regardless of whether they present predominantly with either hepatic injury or neuropsychiatric symptoms [1] or are asymptomatic. The incidence of hepatocellular carcinoma in WD patients with liver cirrhosis is relatively low. A retrospective study of 1,186 WD patients reported that the prevalence of hepatobiliary malignancies in the cohort was 1.2%, and the incidence was 0.28 per 1,000 person-years [1].

### 4.3 Ocular Manifestations

Ocular manifestations of WD commonly include K-F rings, which are usually seen as a golden brown ring in the peripheral cornea caused by the deposition of excess copper on the inner surface of the cornea in the Descemet membrane [1, 4, 32]. K-F rings are present in 90% of WD patients with neurologic manifestations and absent in approximately 60% of patients with hepatic manifestations [1, 22].

Sunflower cataracts occur in 1.2% of newly diagnosed WD patients and are considered a very rare manifestation of WD. These cataracts resemble sunflowers with thin, centralized opacification under the anterior capsule, which is surrounded by secondary opacifications arranged in ray-like structures. Sunflower cataracts do not impact visual acuity and can be reversible after treatment [1, 33].

### 4.4 Hemolysis

Hematological features of untreated WD typically involve hemolysis, which is the result of copper-induced damage to erythrocyte membranes. Severe non-immune intravascular hemolysis that manifests as a sudden and severe drop in hemoglobin is a typical feature of ALF due to WD [1]. The occurrence of thrombocytopenia and leukopenia with hypersplenism may be related to liver cirrhosis and portal hypertension in WD.

In WD patients, Coombs-negative hemolytic anemia occurs as a consequence of high blood copper concentration-induced erythrocyte membrane damage. Hemolytic anemia may occur acutely, paroxysmally, or follow a chronic course. A small-sample study showed that hemolytic anemia was the first manifestation of WD in about 1% of cases and occurred in 28% of WD patients presenting with jaundice. ALF due to WD is often associated with hemolytic anemia.

### 4.5 Other Manifestations

Renal abnormalities in WD patients have been characterized as nephrolithiasis (a potential feature of WD), Fanconi syndrome, hypokalemia, and secondary hypouricemia due to excessive urinary wasting of urate [1, 32]. Kidney injury, primarily renal tubular injury, is manifested by microscopic hematuria and nephrolithiasis. Glomerular injury is the most common complication after long-term chelation therapy. Some WD patients develop heavy proteinuria, which improves and resolves after copper chelation therapy. Patients with rarely observed “osteo-muscular” WD exhibited unique skeletal changes. The clinical manifestations resemble those observed in patients with rickets, including demineralization, renal abnormalities, muscle weakness, and skeletal deformities [1, 22]. Potential cardiac problems associated with WD include cardiomyopathy, arrhythmias, and atrial fibrillation [1, 15, 34]. Endocrine abnormalities in WD patients include hypothyroidism, pancreatitis, infertility, frequent miscarriages in females, gynecomastia, and testicular atrophy [35, 36]. Occasionally, patients with WD develop azure lunula and acanthosis nigricans.

## 5 LABORATORY TESTS

### 5.1 Serum Ceruloplasmin, 24-hour Urinary Copper Excretion, and Serum Copper Tests

The use of a single indicator, such as copper metabolism, often lacks absolute specificity. Combined use of indicators, such as total serum ceruloplasmin (95% reliability), 24-hour urinary copper, and serum copper, should be considered.

#### 5.1.1 Serum Ceruloplasmin

Ceruloplasmin is a copper-carrying protein that is produced in the liver and responsible for the transport of more than 90% of connected copper circulation in healthy individuals. Serum levels are low in newborns, rise gradually with age, and eventually reach a peak in mid-childhood [37]. Therefore, serum ceruloplasmin test should only be performed after 1 year of age, and depend on various districts’ hospitals [12, 22, 38].

WD is characterized by obvious low ceruloplasmin (< 0.1 g/dL) [39]. However, there are several caveats and limitations to the interpretation of ceruloplasmin. First, although the majority (85%) of WD patients with neurological manifestations have a serum ceruloplasmin level of < 0.2 g/L (usually < 0.01-0.1 g/L, or zero sometimes), up to 40% of the patients with active hepatic involvement (including those with ALF) have serum ceruloplasmin levels within the normal range. This is due to the ceruloplasmin, an acute phase protein, being up-regulated in response to liver injury and inflammation. Its level can also be increased by other inflammatory conditions, oral contraceptives, or during pregnancy [40]. Conversely, ceruloplasmin is reduced in copper deficiency caused by malabsorption or zinc supplementation. Up to 30% of heterozygous ATP7B carriers have a mildly reduced serum ceruloplasmin level (0.15-0.19 g/L) [41]. Patients with biallelic mutations in the ceruloplasmin gene located on 3q (not 13q of WD) develop aceruloplasminemia, a very rare neurodegenerative disorder associated with abnormal iron metabolism, anemia, and diabetes [42]. Given all these limitations, the use of ceruloplasmin alone has low diagnostic value for WD. For example, a real-world prospective cohort study involving 2,867 patients with liver disease in secondary care showed that 17 (0.59%) patients had a serum ceruloplasmin level of < 0.2 g/L and only one out of these patients was diagnosed with WD after a comprehensive investigation [43], indicating that the ceruloplasmin had a positive predictive value of only 6% in patients with liver disease.

Ceruloplasmin is the major carrier for copper in the blood, and 90% to 95% of the copper in the blood circulation of normal individuals exists in the form of copper-connected ceruloplasmin (*i.e*., holo-ceruloplasmin). Ceruloplasmin levels are very low in newborns but gradually increase with age and reach adult levels by 1 year of age. Therefore, the youngest age suitable for testing serum ceruloplasmin for the diagnosis of WD is 1 year. The normal range of serum ceruloplasmin is 0.2-0.4 g/L, a level of <0.1 g/L is highly suggestive of WD, a level of 0.1 to 0.2 g/L can be observed in patients with WD, and some heterozygote ATP7B mutation carriers. And particular, about 5% of WD patients do not have reduced ceruloplasmin, or say, normal level. In comparison with WD patients presenting predominantly with liver damage, the reduction in serum ceruloplasmin is more markedly in patients predominantly presenting with neurological symptoms.

Notably, reduced ceruloplasmin can also be found in a variety of disorders other than WD, including liver failure, malnutrition, nephrotic syndrome, protein-losing enteropathy, malabsorption, acquired copper deficiency, glycosylation disorders, Menkes disease, and aceruloplasminemia. Furthermore, ceruloplasmin is an acute phase reactant, and its concentrations can be increased due to acute inflammation and in states associated with hyper-estrogenemia (such as pregnancy, estrogen supplementation, and the use of some oral contraceptives) and liver transplantation.

#### 5.1.2 24-h Urinary Copper Excretion Test

Urinary copper excretion varies throughout the day, so a 24-h urine collection is required. The urine copper samples should be collected into a container that is rinsed with deionized water. 24-h urine collection is time-consuming and inconvenient for some patients and may be difficult.

There is limited data regarding the cutoff value of 24-h urinary copper excretion in adults [27]. In previous guidelines, a higher cutoff value of 1.6 μmol/24 h (100 μg/24 h) is recommended as diagnostic of WD in the main countries, *e.g*., in the USA and China. In a study of 111 healthy adults from the UK, the mean copper excretion was 0.34 μmol/24h (21 μg/24h). Most clinical biochemists consider a copper excretion >0.64 μmol/24h in adults to be abnormal. Other causes of increased urinary copper excretion include cholestatic liver disease, autoimmune hepatitis, non-alcoholic fatty liver disease (NAFLD), and nodular regenerative hyperplasia [44]. Cholestasis inhibits biliary copper excretion, thus causing systemic copper overload and an obvious increase in urinary copper output, especially in children [45].

##### 5.1.2.1 Instructions for 24-hour Urine Collection

1. Empty the bladder to have all urine into the toilet upon awakening and record the date and time as the start time to prepare to collect all 24-hour urine.
2. Collect all urine passed during the day and night into a container.
3. Upon awakening, empty your bladder completely into the container. After 24 hours, record the date and time as the stop time.
4. Return the container with all collected urine and the relevant forms to the Laboratory of the hospital.

24-h urinary copper excretion indirectly reflects the serum-free copper (non-ceruloplasmin-bound copper) levels, which is helpful for diagnosis and treatment monitoring of WD. A 24-h urinary copper excretion greater than 100 μg/24 h has an important diagnostic value for WD; the higher the urinary copper, the better the diagnostic value. Notably, it is difficult to distinguish WD from other liver diseases based on 24-h urinary copper alone because elevated 24-h urinary copper can also be observed in patients with chronic active liver diseases such as autoimmune hepatitis, cholestatic liver disease, and those with ALF of other etiologies. The urinary copper excretion is typically <200 μg /24 h in these patients with liver diseases. Additionally, for WD patients receiving anti-copper therapy, urinary copper is an important reference indicator for assessing treatment efficacy, adherence, or adjusting drug dosage.

The D-penicillamine challenge test has a sensitivity of only 12% and a specificity of up to 96.5% for the diagnosis of WD in asymptomatic children, but its significance for the diagnosis of WD in adults needs to be further clarified.

During the measurement of 24-h urinary copper excretion, the use of plastic containers or acid-washed glass containers is a necessity for the collection of 24-h urine samples. Urinary copper excretion is influenced by 24-h creatinine clearance, so the use of 24-h urinary copper excretion is not recommended for the diagnosis and evaluation of WD in patients with renal failure.

#### 5.1.3 Serum Copper

Serum copper concentrations reflect the levels of both ceruloplasmin-bound copper and non-ceruloplasmin-bound copper. The latter is also known as “free” copper, which is toxic and loosely bound to albumin, small peptides, and amino acids. The decrease in ceruloplasmin-bound copper is proportional to the degrees of hypoceruloplasminemia. Patients with low serum ceruloplasmin levels may have low serum copper, and patients with high serum ceruloplasmin may have high serum copper, regardless of the underlying causes [46]. In WD patients, a combination of low serum ceruloplasmin and normal copper concentrations is often associated with severe acute liver injury and hemolysis. In particular, the calculated non-ceruloplasmin-bound copper concentrations may be abnormally low or zero due to inaccurate ceruloplasmin assays [47, 48].

#### 5.1.4 Copper-65 Absorbance Test

The majority of copper in the body has an atomic weight of 63 Da (^63^Cu), and a small portion has a stable, non-radioactive isotope with an atomic weight of 65Da (^65^Cu). The copper-65 absorbance test involves the administration of 3mg of ^65^Cu in solution, approximately 2-3 times the recommended daily intake of copper (https://www.nhs.uk/conditions/vitamins-and-minerals/others/). The ^65^Cu/^63^Cu ratio is measured in serum samples at baseline and after 1, 2, 6, and 72 hours. A distinct early peak in the ^65^Cu/^63^Cu ratio can be observed in patients with WD, which progressively declines due to a failure to incorporate ^65^Cu into ceruloplasmin. A small initial peak in the ^65^Cu/^63^Cu ratio can be seen in healthy controls and heterozygous carriers, which increases gradually as ^65^Cu is incorporated into ceruloplasmin [49]. Other experts [50] have found that the test is invaluable in difficult cases and suggest that it should be used more widely.

Serum copper is the sum of ceruloplasmin-bound copper and non-ceruloplasmin -bound copper (also named ‘free’ copper), which is called total serum copper. In WD patients, total serum copper is often decreased in proportion to the ceruloplasmin levels. In patients with ALF, the serum copper concentrations may be markedly elevated due to the sudden release of copper from the damaged liver tissue. Serum non-ceruloplasmin-bound copper (or free copper) concentration is calculated using the following formula: (serum copper (μg/L) - ceruloplasmin (mg/L) × 3.15). Free copper concentrations are increased in patients with WD, which can be elevated above 200 μg/L in most untreated patients (normal reference value <150 μg/L). Methods used to measure ceruloplasmin include immunologic methods and enzymatic assay. The turbidimetric immunoassay used in most laboratories cannot distinguish between copper-binding ceruloplasmin and the precursor of ceruloplasmin. The serum ceruloplasmin measured actually includes the precursor of ceruloplasmin, resulting in a high ceruloplasmin concentration, with reduced accuracy of the calculated non-ceruloplasmin-bound copper. Some scholars suggest that the calculated non-ceruloplasmin-bound copper is relatively reliable when the ceruloplasmin is < 100 mg/L. Serum non-ceruloplasmin-bound copper is used primarily for monitoring treatment efficacy in WD but not for diagnosis. Elevated serum-free copper concentrations can also be found in diseases such as chronic cholestasis and copper toxicity. Free copper levels <50 μg/L are suggestive of copper deficiency in the body and can be seen in WD patients who have overdosed after receiving long-term anti-copper therapy. Recently, some researchers have developed a method for direct measurement of free copper concentrations, but it has not yet been popularized clinically.

### 5.2 Genetic Testing and Family Screening

More than 1000 pathogenic mutations of the ATP7B gene have been described up to date, although some variants may have low clinical penetrance. Therefore, to face a series of novel but rare “private family genotypes,” genetic diagnosis should always be corroborated with clinical, biochemical, and radiological findings [3]. Indeed, the absence of two pathogenic mutations does not exclude a diagnosis of WD by clinically routine gene testing [51]. Exome sequencing identified only 88% of WD patients in a cohort from Poland [52]. Genetic testing should not delay the initiation of chelation therapy when other features of WD are diagnostic. It also plays an important role in family screening.

#### 5.2.1 Genetic Testing

The ATP7B gene spans an approximate size of 80 kb, has a coding region of 4.3 kb, and contains 21 exons. Several databases, such as the Human Gene Mutation Database (www.hgmd.cf.ac.uk), provide up-to-date, publicly available information about ATP7B gene mutation. Mutations are predominantly missense, mainly including homozygous and compound heterozygous mutations. In a small minority of patients, only a single heterozygous mutation in the ATP7B gene is identified. For patients with atypical manifestations and high suspicion of WD, detection of hotspot mutations in the ATP7B gene can be performed initially, and for those without positive findings, screening for mutations in the entire coding region of the ATP7B gene and its flanking sequences should be considered.

#### 5.2.2 Family Screening

ATP7B gene mutation testing can be used as a first-line screening method for first-degree relatives of a proband with WD. Siblings of a proband with WD have a 25% chance of having the disease as the recessively genetic model. Other assessments include: medical history and physical examination associated with liver damage and neurologic involvement; liver function tests (including transaminases, albumin, and conjugated/unconjugated bilirubin), serum ceruloplasmin, basal 24-h urinary copper; and slit-lamp examination of K-F ring in the cornea.

### 5.3 Liver Function Tests

Liver biochemical abnormalities are well-recognized but nonspecific features of WD. Elevated transaminases (including AST and ALT) existed in 40-60% of WD patients, usually ranging between 50-200 U/L, which could occur in patients presenting with neurological symptoms alone [13]. In a European cohort of 53 consecutive WD patients, 60% of patients presented with chronic liver disease showed elevated ALT [53], 11-13% of the patients showed elevated bilirubin at the time of diagnosis, and approximately 10% reported a history of clinically overt jaundice [54, 55]. Hyperbilirubinaemia may reflect liver injury, Coombs-negative hemolysis, or a combination of both.

### 5.4 Complete Blood Count and Coagulation Profile

Hematological changes are common in WD, occurring in one-third of WD patients. Coombs-negative hemolysis is a characteristic but underrecognized feature of WD, occurring in 4-10% of cases [54, 56, 57]. Although hemolysis is often accompanied by anemia or manifests as an acute aemolytic syndrome, especially during childhood or adolescence, its clinical course may be chronic. In a retrospective analysis of 321 patients with WD from the UK, 7% of the patients were found to present initially with acute hemolytic crisis [58]. Of these 22 patients, the diagnosis of WD was delayed in 18 (82%) patients, with progressive liver injury and/or neurological deterioration occurring subsequently [56]. The pathogenic mechanisms leading to hemolysis are not fully understood. However, copper toxicity, together with Peroxides and free radicals secondly, may have direct effects on red blood cells, as well as all blood cells, which would explain the occurrence of apparent hemolysis in ALF when excess copper is released during hepatic necrosis.

Thrombocytopenia and leukopenia may mainly occur under conditions of portal hypertension. In a cohort, 63% of the patients with hepatic manifestations and 52% of the patients with neurological manifestations had platelet counts decline markedly. Elevated prothrombin time and international normalized ratio (INR) may occur simultaneously with hepatic dysfunction but are not features of WD [37]. 47% of patients with hepatic manifestations and 65% of those with neurological manifestations had an international normalized ratio of 1.3 or above [53].

### 5.5 Slit Lamp Examination

K-F rings due to copper deposition in the Descemet’s membrane are relatively clinical characteristics of WD and occur in 71-90% of patients with neurological features at presentation and 24-47% of patients with hepatic manifestations [59, 60]. They can occur with systemic copper overload due to cholestasis [61, 62]. K-F rings are often visible to the naked eye as a yellowish-green or golden-brown discoloration at the periphery of each cornea, which, on closer inspection, is distinct from the underlying iris. They always appear initially as a crescent at the 12 o'clock position, followed by the formation of a lower crescent at the 6 o'clock position, and eventually forming a complete ring. If a clinician finds the presence of K-F rings at the bedside, it may take a few seconds to make an initial diagnosis of WD, but the premise is that the clinician actively looks for them. K-F rings may be undetectable at the bedside, so slit lamp examination by an experienced ophthalmologist may be necessary to confirm or exclude the presence of K-F rings when WD is suspected. If it is difficult to conclude about the presence or absence of K-F rings, anterior segment optical coherence tomography may be helpful [63]. Sunflower cataracts are visible to the naked eye and do not require slit lamp examination to detect their presence.

### 5.6 Penicillamine Challenge Test

Historically, measurement of urinary copper excretion after penicillamine administration (penicillamine provocation test) had been used in the diagnosis of WD, especially in children. However, due to inconsistent dosing and timing of penicillamine, the test results have so far proved unreliable across both Western countries and China.

## 6 LIVER ULTRASOUND

Hepatic steatosis manifests as enhanced liver echogenicity on ultrasound or cross-sectional imaging, and it is a common finding in WD, occurring in 35-88% of patients [64, 65]. However, this is highly nonspecific across a range of other more common conditions, including alcohol-related liver disease and NAFLD. Ultrasound plays an important role in staging the severity of liver disease, which should be performed in any patient with suspected WD and abnormal liver function tests, thrombocytopenia or clinical features of chronic liver diseases. Cirrhosis may manifest as irregular liver margins with or without manifestations of portal hypertension, including reversed portal vein flow, splenomegaly, and ascites. Cross-sectional imaging with CT or MRI may also reveal intra-abdominal collaterals or varices, suggesting elevated portal pressure. Even patients presented only with neurological symptoms have a high rate of liver abnormalities on imaging [53]. Certain radiological features are suggested to partly be specific to WD, including multiple hyperechoic and hypoechoic nodular lesions, a periportal fat layer, and the absence of caudate lobe hypertrophy in cirrhosis. However, these have only been described in case reports and should not be considered finally diagnostic [66].

Liver stiffness measurement (LSM) using transient elastography can be used as an additional tool for non-invasive fibrosis staging in WD, which can be applied in combination with clinical assessment, laboratory investigations, and liver imaging. An LSM cut-off of ≥ 9.9 kPa has good accuracy in identifying cirrhosis in adults with newly diagnosed WD. In the majority of treated WD patients, LSM remains stable over time, and therefore, routine monitoring is not essential [67]. However, transient elastography may be useful in patients with clinical evidence of disease progression or those with poor treatment adherence or response.

## 7 NEUROIMAGING

Specialists need to consider whether a brain MRI is required and whether any previous neuroimaging is consistent with the WD condition. High signal intensity on T2-weighted and fluid-attenuated inversion recovery sequences, often referred to as “lesions,” are typically seen in the bilateral basal ganglia, thalamus, and/or brainstem in patients with neurological manifestations, which can also occur in the white matter of the brain [68-70]. Atrophy of the cerebral cortex, brainstem, and/or cerebellum is also common at diagnosis. The vast majority (90-100%) of WD patients with neurological manifestations can have at least one of these abnormalities, although these findings are not rare in WD patients with hepatic manifestations and those identified by family screening without any nervous symptoms. Depending on the involvement of different nuclei in the basal ganglia, the lesions show signs like “woodpecker,” “figure eight,” “double figure eight,” and “butterfly with wings”, and MRI enhanced scan does not show obvious enhancement of the lesion. Lesions in the posterior midbrain (tectal plate), pons, or simultaneously involving both the basal ganglia and brainstem appear to be highly specific for WD, among other causes of movement disorders [71], as well as central pontine myelinolysis (CPM). A less common but unique feature of WD is the characteristic “Face of the Giant Panda” sign on brain MRI, including high T2 signal intensity in the midbrain [18]. Susceptibility-weighted imaging is often abnormal. High signal intensity in the basal ganglia on T1-weighted images can occur with cirrhosis of any cause. Neurologists should also suggest that WD rarely results in confluent white matter abnormalities, the same as neuroimaging of leukodystrophy. Given that thalamic and brainstem lesions are related to a paradoxical worsening of neurological symptoms in WD patients, neuroimaging findings have important prognostic implications [72, 73]. They can also be seen in patients with hepatic manifestations, 20% of whom go on to develop neurological symptoms that may subsequently require MRI [13]. MRI with 7 Tesla and QSM techniques for WD patients has been studied in China and European countries.

## 8 LIVER BIOPSY

When the diagnosis is uncertain, measurement of hepatic parenchymal copper concentration by liver biopsy may confirm the diagnosis of WD in children and adults. Additionally, liver histology may be helpful in staging the severity of liver disease and ruling out alternative or comorbid pathologies [1]. In the early stage of WD, macrovesicular steatosis may be the only histological finding and can be seen in 70% of cases. Portal inflammation and fibrosis, similar to autoimmune hepatitis, are other histological patterns of WD, although they are typically associated with steatosis. Vacuolated nuclei, degenerated hepatocytes, inflammation, and Mallory-Denk bodies may also be observed. Over time, fibrosis may accumulate and eventually evolve into cirrhosis, which is identified in up to 50% of patients at the time of diagnosis using biopsy [57]. However, these histological features are not specific to WD. Copper and copper-associated proteins can be identified by histochemical staining prior to cirrhosis and are typically located in the periportal areas, with the presence of irregular deposition within and between cirrhotic nodules.

Given the diagnostic challenge in the interpretation of liver morphology, the measurement of hepatic parenchymal copper concentrations is an important part of the liver biopsy workup in WD. Therefore, a portion of the sample should be reserved for this purpose. Normal hepatic copper content in the liver is <50 µg/g of dry weight; a cut-off of ≥250 µg/g has traditionally been regarded as diagnostic of WD. However, in a large prospective diagnostic accuracy study in 3,350 consecutive patients undergoing liver biopsy, this cut-off value has been revised downward to 209 µg/g; 178 out of these patients were ultimately diagnosed with WD [2, 74].

Furthermore, copper staining is non-specific for WD and observed in a range of other cholestatic liver diseases, including primary biliary cholangitis, biliary atresia, prolonged extrahepatic biliary obstruction, primary/secondary sclerosing cholangitis, and heterozygous carriers of the ATP7B gene variant. In terms of the method of liver biopsy, percutaneous puncture is preferred, but if there is concern for the risk of hemorrhage after biopsy, a transjugular approach should be considered.

### 8.1 Brain Imaging

MRI is more sensitive than CT for detecting brain lesions. If left untreated, brain MRI changes can appear in almost 100% of patients with neurological WD, 40-75% of patients with hepatic WD, and 20-30% of asymptomatic patients. Also, others refer to Paragraph 7.

### 8.2 Liver Imaging

WD with liver involvement is demonstrated by diffuse damage. In some patients, there are multiple nodules in the liver, which show high signal intensity on plain CT, high signal intensity on T1-weighted images, and low signal intensity surrounded by high signal intensity septa on T2-weighted images, presenting as a relatively characteristic honeycomb pattern. These nodules are thought to be hepatic regenerative nodules surrounded by fibrous septa. The “honeycomb pattern” is useful for differential diagnosis but has limited diagnostic sensitivity and is not specific for WD. The use of positron emission tomography with radioactive copper as a tracer for assessing liver involvement in WD patients has also been tested in clinical trials, and the results obtained are positive and reliable [75].

## 9 LIVER PATHOLOGY

Liver pathology can be performed when WD is clinically suspected or other liver diseases need to be excluded. It is helpful for histological diagnosis of WD, determination of the degree of lesions, and evaluation of treatment efficacy. The pathological diagnosis of WD is mainly based on copper deposition within the hepatocytes, which is characterized by inflammation of varying severity and fibrosis of varying degrees according to the degree of lesions and different stages of disease progression. Early lesions are mild and nonspecific, with the visualization of only focal necrosis, the formation of apoptotic bodies, hepatocellular ballooning degeneration, hepatocellular steatosis, and hepatocellular glycogenated nuclei that are typically localized in acinar zone 1. Additionally, mild portal inflammation with primary lymphocyte infiltration and rare interface dermatitis may be observed. Lobular inflammation, necrosis, and portal inflammation worsen as WD progresses. Some patients with WD show lesions similar to those of steatohepatitis, including marked hepatocellular steatosis, hepatocytic ballooning, Mallory-Denk body formation, and necrotic foci with infiltration of a mixed population of inflammatory cells. Some patients present with changes mimicking chronic hepatitis, including portal inflammation with interstitial infiltration of many lymphocytes and few plasma cells interface dermatitis, with periportal ductular reaction. The end-stage WD is suggested by macronodular and mixed macro-and micronodular cirrhosis. A small number of WD patients have massive confluent hepatocellular necrosis, exhibiting pathological changes associated with ALF and acute-on-chronic liver failure. Some cases of WD along with Liver carcinoma are observed, but not lower than had been suspected.

### 9.1 Recommendations

1. Screening for WD should be considered in patients at any age, especially in adolescent and young adult patients who present with unexplained neuropsychiatric symptoms or abnormal liver function test results (1A).
2. For patients with suspected WD, K-F rings should be detected using slit lamp examination by an experienced ophthalmologist (1B).
3. WD should be highly suspected with serum ceruloplasmin < 100 mg/L; the diagnosis of WD should not be excluded when the ceruloplasmin concentrations are within the normal range or critical values (1A); and the possibility of WD should be excluded when the serum ceruloplasmin concentrations are above the upper limit of normal range (2A).
4. Basal 24-h urinary copper >100 μg/24 h is considered diagnostic for WD in symptomatic patients (1A).
5. Adolescents and young adults with Coombs-negative hemolytic anemia should be screened for WD (2C); acute severe hemolysis can be an initial manifestation of ALF due to WD (2C).
6. ATP7B gene mutation detection is recommended to confirm the diagnosis in patients with suspected WD (1B). ATP7B gene mutation detection is recommended as the first-line screening method for family screening, especially when ATP7B gene mutations are identified in a proband with WD (1B); first-degree relatives of a proband with WD should be screened (1A).
7. Brain MRI can be used as a means to assess conditions and monitor treatment efficacy in WD patients presenting with neurological symptoms (1A). Routine MRI 3 T is enough; in special cases, 7 T and QSM might be recommended in China.

## 10 DIAGNOSIS

WD should be considered when patients present with one or more of the following symptoms: 1) liver abnormalities of uncertain cause, regardless of age; 2) unexplained liver disease associated with neurological or psychiatric disorders; 3) ALF with nonimmune hemolytic anemia, including acute intravascular hemolysis; 4) recurrent self-limited nonimmune hemolysis [1].

Patients with cirrhosis, neurological manifestations, and K-F rings are relatively easy to diagnose because they present with overt clinical signs and symptoms. However, approximately half of the patients presented with liver disease do not possess two of these three criteria. It is, therefore, challenging to establish a diagnosis [1, 9].

Neurological symptoms of WD appear later than liver abnormalities but are typically the first clinical symptoms leading to the diagnosis. Approximately 68% of patients develop initial neurological symptoms. The mean age at onset of symptoms is 20-30 years, but a wide range (6-72 years) of presentations of new neurological symptoms is observed [6].

Once WD is considered, a detailed personal and family history should be collected, and a physical examination focusing on evidence of hepatic, neurological, and psychiatric disorders should be performed, so a combination of assessments is recommended [1].

Generally, serum ceruloplasmin is usually markedly low in WD, with a value of <0.12 g/L (normal reference value 0.2-0.4 g/L). However, the predictive value in this setting is poor. Low ceruloplasmin can occur in patients without WD, and ceruloplasmin within the normal range does not completely exclude a diagnosis of WD. Therefore, relying only on serum ceruloplasmin levels alone is not sufficient to establish a diagnosis [1].

Non-ceruloplasmin-bound copper levels may be a representation of actual organ damage and a valuable diagnostic and detection tool; however, their reliable measurement is currently not clinically available. Measurement of the amount of copper excreted in the urine in a 24-hour period (basal 24-h urinary excretion) is an important part of WD diagnosis, as it reflects the amount of non-ceruloplasmin-bound copper in the circulation. Basal 24-h urinary copper excretion for symptomatic patients with WD is typically >100 μg/24 h (>1.6 μmol/24 h). A basal 24-h urinary copper excretion of >40 μg/24 h (>0.6 μmol/24 h) might be suggestive of WD in asymptomatic individuals or children and, therefore, requires clinical correlation and further investigation. The use of a multidisciplinary approach is advocated for some assessments. Specifically, assessment of the eyes with a slit lamp for eye examination requires a skilled observer and detection of neurological signs or symptoms requires referral to a neurologist or movement disorder specialist [1].

WD patients, especially younger patients, may have clinical features and histologic findings on liver biopsy that are indistinguishable from autoimmune hepatitis. WD should be excluded in children with overt autoimmune hepatitis and those with autoimmune hepatitis not responding or only partially responding to corticosteroid therapy. In rare cases, autoimmune hepatitis and WD may coexist [1, 76-78]. The diagnosis of WD may be particularly difficult in patients with multiple etiologies of chronic liver disease, including NAFLD and alcohol-related liver disease. It is important to maintain a thorough differential diagnosis for patients with an established diagnosis of WD and more obvious etiologies of liver disease. WD patients may also develop symptoms of multiple etiologies of liver injury. Hepatic steatosis in WD can be as severe as in NAFLD and, in some cases, nonalcoholic steatohepatitis. Basal 24-hour urinary copper excretion and hepatic copper concentrations may be low in patients with NAFLD. This can help differentiate WD from NAFLD [1, 79].

Genetic analysis is not required for the diagnosis of WD, but it is useful in confirming WD, especially in difficult cases without clear clinical manifestations, and facilitating subsequent screening of family members. A molecular genetic strategy using direct mutation analysis may be particularly effective in identifying affected siblings of a proband with 2 identified ATP7B mutations [1, 18]. Considerations for genetic testing include availability and cost. Patients with inconclusive genetic testing results need to undergo clinical and biochemical assessment [1].

### 10.1 Guidance for WD Diagnosis in Patients with Liver/ Gastrointestinal Disease

1. Asymptomatic patients without organ damage: low or mildly low ceruloplasmin (between 14 and 20 mg/dL); mildly increased 24-h urinary copper excretion (>40 μg/24 h); increased non-ceruloplasmin-bound copper; and increased hepatic copper levels (>75 μg/g).
2. Patients with hepatitis-mimic: low or normal ceruloplasmin; increased 24-h urinary copper excretion (>100 μg/24 h); increased non-ceruloplasmin-bound copper; and increased hepatic copper levels (>250 μg/g).
3. Patients with steatosis: low ceruloplasmin (<14 mg/dL); increased 24-h urinary copper excretion (>100 μg/24 h); increased non-ceruloplasmin-bound copper; and increased hepatic copper levels (>250 μg/g).
4. Patients with cirrhosis: low or very low ceruloplasmin (<5 mg/dL); increased 24-h urinary copper excretion (>100 μg/24 h); increased non-ceruloplasmin-bound copper; increased hepatic copper levels (>250 μg/g); thrombocytopenia; increased international normalized ratio.
5. Patients with ALF: ALP: TB ratio < 4, AST:ALT ratio = 2.2; low or elevated ceruloplasmin; increased non-ceruloplasmin-bound copper; increased 24-h urinary copper excretion (>100 μg/24 h); and increased hepatic copper levels (>250 μg/g). Hemolysis is suggested by decreased haptoglobin levels, Coombs-negative hemolytic anemia (high reticulocyte count); low hemoglobin may be present if hemolysis occurs; low white blood cells and thrombocytopenia appear in patients with hypersplenism due to portal hypertension; increased international normalized ratio.

The Leipzig score is helpful for diagnosing WD. This arithmetic scoring system includes clinical and biochemical findings and has been validated in children and adults. Prognostic scoring systems are used to predict when a patient with WD will fail to respond to treatment. The first prognostic scoring system is the Nazer score, which is based on serum bilirubin, AST, and prothrombin time [1, 80]. The Nazer score was superseded by the New Wilson index, with white blood cell count, serum albumin, and INR (not prothrombin time) being added.

Since there is currently no optimal diagnostic scoring system, this consensus recommends the use of the diagnostic criteria (Leipzig scoring system) developed at the 8^th^ International Meeting on Wilson Disease held in Leipzig in 2001 to establish a diagnosis of WD. The diagnosis of WD is highly likely with a total score of ≥ 4, probable with a score of 3, and unlikely with a score of <2.

Leipzig score has clinical utility and is convenient. Considering that genetic testing has been gradually popularized and adopted in clinical practice, a live biopsy is invasive, and liver copper distribution is often uneven, it is recommended to calculate the Leipzig score at each step and follow the diagnostic process shown in Fig. (**1**), once a total score of ≥ 4 points is obtained, WD diagnosis can be confirmed, and treatment should be initiated.

## 11 DIFFERENTIAL DIAGNOSIS

### 11.1 Differentiation of WD Clinically from Other Related Diseases

1. For patients presenting with neuropsychiatric symptoms as the main manifestations, WD should be differentiated from disorders such as early-onset Parkinson's disease or other causes of parkinsonism, various etiologies of dystonia, chorea, primary tremor, various kinds of extrapyramidal symptoms, other causes of psychiatric abnormalities, and epilepsy.
2. For patients who present predominantly with acute and chronic hepatitis-mimic, liver failure, or cirrhosis, WD should be differentiated from other disorders causing hepatitis, liver failure, and cirrhosis, such as viral hepatitis, alcoholic-related liver disease, autoimmune liver disease, and drug-induced liver injury.
3. For patients presenting with hemolytic anemia as the main manifestation, WD should be differentiated from other diseases that cause hemolysis and anemia. Acute hepatitis and hemolysis due to WD during pregnancy should be differentiated from hemolysis, elevated liver enzymes, and low platelet count syndrome.
4. If patients primarily present with symptoms of other visceral systems, WD should be differentiated from corresponding diseases according to specific conditions. For example, if the primary manifestation is kidney injury, WD should be differentiated from other common diseases causing kidney injury, such as nephritis or kidney disease.

### 11.2 Differentiation of WD from Other Inherited Metabolic Disorders

Chronic cholestatic liver disease, disorders of copper (manganese) metabolism, and congenital disorders of glycosylation can manifest with hepatic and/or neurological symptoms, with one or more copper metabolism-associated indicators being abnormal, such as decreased serum ceruloplasmin, elevated 24-h urinary copper excretion, and excessive copper deposition in the liver. Attention should be paid to the differential diagnosis in patients without a confirmed diagnosis of WD or those with clinically diagnosed WD but a poor response to anti-copper treatments, for example, Menkes disease should be considered in the differential diagnosis of WD. The authors reported a group of patients with Brain iron deposition and whole-exome sequencing of non-Wilson's disease hypoceruloplasminemia [47].

### 11.3 Recommendations

1. The Leipzig scoring system is recommended for the diagnosis of WD. Leipzig score can be calculated in a stepwise manner according to the order of the tests, including clinical manifestations, biochemical, genetic testing, and histological examination of liver biopsy. Once a total score of ≥ 4 is obtained, the diagnosis can be confirmed and treatment can be initiated (1A).
2. A combination of AST: ALT ratio > 2.2 and ALP: TB ratio < 4 is shown to be predictive with respect to ALF due to WD, and the diagnostic process for WD should be initiated in such patients immediately (1B).

## 12 TREATMENT AND MONITORING

Lifelong medical treatment is indicated in all patients with newly diagnosed WD. The primary treatment for WD is copper-directed and includes pharmacologic therapy that chelates copper and blocks copper absorption [1]. Failure to comply with lifelong therapy usually leads to recurrent or the onset of new symptoms (including neurologic or psychiatric and hepatic symptoms), liver failure, ultimately resulting in the need for liver transplantation, and the new onset or worsening of neuropsychiatric symptoms [1].

A personalized dietary plan should be created to maintain a dietary copper intake of <0.9 mg/day. Dietary counseling should complement pharmacologic therapy. Initial treatment of symptomatic patients with WD should include chelating agents [1]. Experts from Europe and America suggest that Trientine may be better tolerated than D-penicillamine [1]. Chelating agents act by chelating copper and promoting urinary copper excretion, which is easier to identify by urinary copper concentration. D-penicillamine is the first oral chelating agent used to treat WD and is considered one of the mainstays of therapy for WD in the Western countries. D- penicillamine can produce many adverse effects, including worsening of neurological symptoms, leading to treatment discontinuation in nearly a third of patients with WD. Toleration of D-penicillamine may be enhanced by starting with gradually incremental doses, and of course, the “low and slow” approach has not been studied in controlled trials [18, 22]. Dimercaptosuccinic acid (DMSA) capsules are commonly used to treat WD patients in mainland China. They are also used in some Western & other countries (only treated by informal private WD patients themselves in California or other states USA) but have not been widely accepted due to a lack of randomized controlled trials. The use of DMSA capsules for the treatment of WD can also be found in Singapore, Hong Kong, and China.

Trientine has the same mechanism of action as D-penicillamine, which is particularly indicated for patients with D-penicillamine intolerance [1, 2, 18]. In 2022, oral trientine tetrahydrochloride was approved as a maintenance therapy for adults with stable WD who are tolerant to penicillamine and approved in China in 2024. The approval of trientine tetrahydrochloride was based on the randomized, open-label, non-inferiority, phase 3 trial (CHELATE) [1, 2, 18]. Researchers demonstrated that trientine tetrahydrochloride is non-inferior to penicillamine in maintaining serum non-ceruloplasmin-bound copper levels. Furthermore, the clinical trial provides data on specific serum copper bound to ceruloplasmin *versus* other serum components, proposing a test of potential clinical validity to monitor labile copper levels. Trientine appears to have fewer adverse effects than D-penicillamine, including paradoxical neurologic deterioration after initiation of treatment [1, 2, 18]. When D-penicillamine is replaced with trientine tetrahydrochloride, the adverse effects caused by D-penicillamine tend to resolve, and do not recur. However, neurologic deterioration due to D-penicillamine may not resolve after switching from D-penicillamine to trientine tetrahydrochloride. These observations are based on accumulated clinical experience. A head-to-head comparison between D-penicillamine and trientine for the initial treatment of WD has never been performed [1, 2, 18]. The authors further observed that a few Chinese WD patients had safely been carried out by trientine-related RCT clinical test by PI: Brewer GJ some decades years ago [22].

Treatment of asymptomatic WD patients is based on chelating agents (D-penicillamine or trientine at a lower dosage than that used for initial treatment), zinc, or Gandou tablet (a traditional Chinese medicine that has been used in the treatment of WD, mostly in China) [22]. All asymptomatic patients require treatment, but the urgency of treatment is greater if organ damage is evident. Zinc has traditionally been reserved for maintenance therapy in WD, which is used commonly and is now recommended by the AASLD as first-line therapy for asymptomatic patients [18, 22]. Zinc can also be considered a first-line alternative treatment as well as a maintenance treatment for patients with prevalent neurological manifestations. Its slow action and mechanism of action are typically not associated with paradoxical worsening of neurological signs and symptoms. Zinc inhibits intestinal copper uptake by inducing the synthesis of enterocyte metallothionein (an endogenous chelator of metals) [1]. Copper is mainly excreted into the feces, resulting in a negative copper balance. Zinc also reduces hepatic copper toxicity by inducing metallothioneins in hepatocytes, which can chelate copper in a nontoxic form or a form that is less prone to oxidative stress. In the case of zinc treatment, urinary copper levels will be low, which is an indicator of treatment adherence or adequacy [22].

WD is an inherited metabolic disease that can be successfully treated with medicines, and the long-term prognosis of WD patients depends on whether treatment is initiated earlier or later relative to disease onset. The earlier the treatment, the less the damage and the better the prognosis. Appropriate anti-copper and protective treatments may help WD patients achieve an almost normal lifespan [22]. Once the WD diagnosis is confirmed, the initial pharmacologic therapy should be started as soon as possible, but it is different from steroid pulse therapy [22]. There are great individual differences in response to medications among WD patients. Currently, there are no medications available for the treatment of all WD patients, and the most appropriate treatment regimens should be selected according to the conditions of each patient: “Start low dose and have slow step” [18, 22]. Anti-copper therapy cannot correct genetic defects in patients. Even if the treatment efficacy is good, this treatment should not be stopped. Discontinuation of the treatment results in recurrence and deterioration of symptoms and even liver failure [1, 18, 22].

Recent studies have demonstrated that approximately 80% to 85% of treated patients with WD have a good long-term prognosis. Physicians and patients should have sufficient confidence in the prognosis of this disease. Patients with severe conditions are not contraindicated for anti-copper therapy. Conversely, if the condition is more severe, more rapid treatment may be required.

Various problems may occur during the treatment of WD patients, which can lead to serious consequences if not identified and treated in a timely fashion. Therefore, the treatment efficacy, adverse effects, and adherence must be regularly monitored during treatment.

Treatment for WD is generally divided into (1) Initial treatment and (2) Maintenance therapy [2, 18]. (3) Steroid-like Pulse therapy is administrated partly in suitable patients in mainland China. Generally, patients’ symptoms or biochemical abnormalities tend to stabilize within 6 to 12 months of initial treatment in China. Thereafter, Pulse therapy is managed [22]. It is worth noting that the initial treatment may worsen the neurological symptoms and hepatic manifestations [1, 18, 22]. Therefore, a personalized management strategy for pulse therapy should be carefully considered [22].

### 12.1 Pharmacologic Therapy

Drugs used in the treatment of WD are divided into two main categories: chelating agents that promote urinary copper excretion and drugs that block copper absorption. Although these two classes of medicines have different mechanisms of action, they can both reduce copper accumulation in the body, thus achieving “physiological” copper balance.

### 12.2 Drugs that Increase Urinary Copper Excretion

#### 12.2.1 D-penicillamine

D-penicillamine was first introduced as the oral drug for treating WD in 1956, which presently is the first choice for patients with WD in developing countries [1, 18, 22]. D-penicillamine is absorbed rapidly, with a bioavailability between 40% and 70%. It is mostly metabolized in the liver, greater than 80% of the drug and its metabolites are excreted *via* the kidneys, with an excretion heal-life of 1.7-7.0 h. D-penicillamine chelates copper through a sulfhydryl group and promotes the excretion of copper *via* urine. It also induces metallothionein production in hepatocytes and reduces the hepatotoxicity of copper after binding with copper. Individual response and tolerance to D-penicillamine vary greatly among patients with WD, so personalized treatment regimens should be developed for each patient according to their conditions. D-penicillamine can hardly cross the blood-brain barrier and, therefore, is more likely to induce the occurrence and worsening of neuropsychiatric symptoms [1, 18, 22].

##### 12.2.1.1 Indications

D-penicillamine is indicated for patients with various clinical forms of WD. Given the high risk of neurological deterioration following D-penicillamine treatment, it should, therefore, be used with caution in patients with severe neurological symptoms.

##### 12.2.1.2 Dosing and Administration

1. Children: 125-250 mg/day, increased slowly by dose increments of 125-250 mg/week to an initial target dose of 20 mg/kg/day in two divided doses carefully (maximum 1500 mg/day) [18, 22]; 2000-3000 mg/day had previously proposed by experts from the Western countries) [22].
2. Adults without neurologic or psychiatric symptoms: Initial target dose of 1000-1500 mg/day in two divided doses.
3. Adults with neurological or psychiatric symptoms: 125-250 mg/day, increased slowly by dose increments of 125-250 mg/week to an initial target dose of 1000-1500 mg/day very carefully.
4. Maintenance dose (typically after 2 years): 10-20 mg/kg/day in two divided doses.

D-penicillamine can be administered to patients only when the negative penicillin skin test. The tolerability of D-penicillamine may be enhanced by starting with a low dose. For adults, the initial dose is 125-250 mg/d, increased by 250 mg/d increments every 4 to 7 days to a maximum dose of 1,000 to 1,500 mg/d, and the maintenance dose is 750 to 1,000 mg/d (or 10 to 15 mg/kg/d), administered in 2-4 divided doses. For children, lower doses can be used initially and gradually increased to 20 mg/kg/d (maximum 750-1,000 mg/d). The maintenance dose is 10-20 mg/kg/d. Food can affect the absorption of D-penicillamine. It should, therefore, be administered either 1 hour before or two hours after a meal. Since D-penicillamine can interfere with vitamin B_6_ metabolism, vitamin B_6_ (10-30 mg/d) should be supplemented simultaneously [18, 22].

##### 12.2.1.3 Evaluation of Treatment Efficacy

For WD patients presenting with liver disease as the main manifestation, liver function usually improves markedly after 2-6 months of treatment, and complete remission can be achieved 1 year after treatment. For patients presenting with neurological symptoms, their symptoms and signs improve slowly, and it often takes 1 to 3 years for a patient to recover. During treatment, the patient’s symptoms and signs, blood routine and urine routine results, liver and kidney function, 24-h urinary copper excretion should be observed and detected regularly. In general, evaluation can be performed once or twice a month after starting treatment, once every 1 to 3 months after liver function is improved, and 2 to 3 times a year during maintenance therapy. 24-h urinary copper is a useful indicator to assess treatment efficacy and adherence. 24-h urinary copper often reaches a peak after 1 month of initial treatment, up to 1,500-8,000 μg/24 h, and then gradually decreases. After 6 months to 1 year of treatment, 24-h urinary copper can reach its target range on maintenance therapy, *i.e*., 200-500 μg/24 h, in most patients, but the time required varies greatly across individuals. Urinary copper >100 μg/24 h after 48 h of D-penicillamine cessation indicates poor treatment adherence [1, 2].

##### 12.2.1.4 Adverse Effects

Early reactions include allergic reactions (such as fever and rash), proteinuria, myelosuppression (neutropenia, thrombocytopenia), smell and taste disorders, and paradoxical neurological worsening. Later reactions include lupus-like syndrome, Goodpasture's syndrome, elastosis perforans serpiginosa, and poor wound/surgical incision healing [1, 2, 22].

Adverse reactions are more frequent after D-penicillamine treatment, leading to treatment discontinuation in approximately 30% of patients. Allergic reactions characterized by fever, skin rash, and lymphadenopathy, as well as proteinuria, neutropenia, and thrombocytopenia, commonly occur 1-3 weeks after starting the treatment. D-penicillamine should be discontinued immediately. For patients who experience mild fever and rash, anti-allergy treatment and desensitization should be given, such as oral prednisolone at a low dose of 0.5 mg/kg/day for 2-3 days. After patients’ symptoms are relieved, D-penicillamine treatment can be restarted at a low dose (*e.g*., 5 mg/kg/d), which is gradually increased. The dose of corticosteroids is reduced gradually and discontinued. Worsening of neurological symptoms can occur in 10-50% of patients after beginning treatment with D-penicillamine [1, 2, 22].

Nephrotoxicity is one of the commonly observed adverse effects following treatment with D-penicillamine, presenting as proteinuria and/or hematuria and occasionally acute renal failure. In such cases, D-penicillamine must be discontinued immediately. Skin toxicity includes degenerative changes of the skin, elastosis perforans serpiginous, pemphigus or lichen planus pemphigoid, and recurrent aphthous stomatitis. If neutrophilia and thrombocytopenia occur during treatment, it is necessary to determine whether these events are caused by laboratory error, hypersplenism, or adverse drug reactions. Additionally, hepatotoxicity can also be seen in patients after treatment with D-penicillamine, but there are no definitive diagnostic criteria for hepatotoxicity. Its incidence is difficult to estimate.

The adverse effects of D-penicillamine do not parallel disease severity. The severity of the disease is not a contraindication to the use of D-penicillamine. Close observation, early detection, timely dose reductions, and treatment cessation are the only ways to prevent serious consequences of adverse effects of D-penicillamine, especially in countries of the Third World [1, 2, 22]. On the other hand, oral DMSA is available in China, while Trientine is available in the West for specific situations.

#### 12.2.2 Sodium Dimercaptosulphonate (DMPS) Intravenously

DMPS was originally developed by a scientist in the former Soviet Union, which is a heavy-metal chelating agent with 2 sulfhydryl groups and good water solubility [22]. DMPS can obviously promote the excretion of heavy metals. It was first used for the treatment of WD in China. The copper-chelating effect of DMPS is 2.6 times of D-penicillamine (calculation based on the use of 750 mg of DMPS and 1,000 mg of D-penicillamine). The adverse effects, such as worsening of neurological symptoms, are less commonly seen after DMPS treatment compared with D-penicillamine treatment. Prof. Yang RM systematically studied the use of DMPS in the treatment of WD and has promoted its application in China for 50 years [22].

##### 12.2.2.1 Indications

DMPS is considered to be used in patients with WD presented with severe conditions (such as ALF) and neuropsychiatric symptoms, as well as those who are allergic to D-penicillamine or those who are not effectively treated by D-penicillamine and require rapid copper reduction. DMPS can be used in combination with zinc or used alternately with D-Penicillamine, traditional Chinese medicinal herbs, and zinc [22].

##### 12.2.2.2 Dosing and Administration

The adult dose is 500-750 mg per day. DMPS dissolved in 500 ml of 5% dextrose is administered *via* intravenous infusion once a day, with 5 consecutive days as a course of treatment and an interval of 2 days between the two courses. Multiple treatment courses can be performed. The dose of DMPS is 10 mg/kg/d for children (maximum 750 mg/d) [22].

##### 12.2.2.3 Evaluation of Treatment Efficacy

24-h urinary copper often reaches a peak after 1-2 weeks of initial treatment, up to 2,000-10,000 μg/24 h, and then gradually decreases in 3-4 weeks [22].

##### 12.2.2.4 Adverse Effects

Rapid intravenous infusion may result in symptoms such as nausea, tachycardia, and dizziness. They are alleviated by slowing down the infusion rate. Some patients experience allergic reactions such as rash, chills, and fever, which are generally mild and disappear soon after discontinuation of DMPS treatment. Intravenous infusion of calcium gluconate immediately after DMPS treatment can reduce the occurrence of adverse reactions. Treatment should be paused during menstruation and gastrointestinal bleeding [22].

#### 12.2.3 DMSA Capsules

DMSA is a broad-spectrum heavy-metal chelating agent developed by Prof. Ding GS in China [22]. Its mechanism of action is that the two active sulfhydryl groups in its molecules can bind with copper in tissues to form stable, water-soluble chelated compounds, which are then excreted in the urine, especially by the stool. DMSA has been studied and used systematically by the team led by Prof. Yang RM and has been used as the first-line drug for WD in China [22]. DMSA exerts a weaker copper-chelating effect than D-penicillamine, but it is lipid soluble and can cross the blood-brain barrier, thus facilitating the improvement in the neuropsychiatric symptoms, with relatively few adverse effects. It has already received much international attention, with generic drugs being created and used in some countries throughout the world [22].

##### 12.2.3.1 Indications

DMSA is indicated in WD patients with varying degrees of liver injury or neuropsychiatric symptoms, as well as those who are allergic or intolerant to D-penicillamine. It can be used in combination with zinc or used alternately with traditional Chinese medicinal herbs and D-penicillamine in China [22].

##### 12.2.3.2 Dosing and Administration

DMSA is an oral capsule preparation. The dose is 750-1,000 mg/d for adults and 10-20 mg/kg/d for children, administered orally in 2 divided doses. DMSA can also be used for long-term maintenance treatment [22].

##### 12.2.3.3 Evaluation of Treatment Efficacy

On the first day of DMSA treatment, urinary copper levels in patients often increase by about 100-300 μg/24 h compared with those before treatment, which reaches a peak within 1 month after treatment and decreases slowly after continuing treatment.

##### 12.2.3.4 Adverse Effects

DMSA has fewer adverse effects, mainly including mild gastrointestinal symptoms, such as nausea, vomiting, abdominal distension, loss of appetite, and halitosis. Rash, itching, transient thrombocytopenia and transaminase elevation can occur in a few patients [22].

#### 12.2.4 Trientine

Trientine is a metal ion chelating agent and has a polyamine-like structure. Its gastrointestinal absorption rate is very low. Approximately 1% of the trientine and 8% of its metabolites can be excreted *via* the kidneys. Old-type double-hydroxyl Trientine product is unstable and should be stored tightly closed at 2-8°C. However, trientine *tetrahydrochloride* (Cuprior) is stable in the normal environment. Trientine has replaced D-penicillamine as the first choice for WD in Europe and America [1, 2, 18]. Presently, it has been approved by China FDA since 2024 Spring https://www.bjnews.com.cn/detail/1704585025168315.html.

##### 12.2.4.1 Indications

Trientine can be used in patients with all clinical forms of WD, especially those with neuropsychiatric symptoms and those who are allergic or intolerant to D-penicillamine. Iron supplementation should be avoided during trientine treatment as copper-iron chelates can produce toxic complexes [1, 2, 18].

##### 12.2.4.2 Evaluation of Treatment Efficacy

24-h urinary copper excretion reaches a peak within 1-2 months after initial treatment with trientine and then decreases slowly.

##### 12.2.4.3 Adverse Effects

Adverse effects due to trientine include urticaria/rash, arthralgia/myalgia, proteinuria, haematuria, sideroblastic anaemia, and paradoxical worsening of neurological symptoms.

It is initially believed that the adverse effects of trientine are rare. However, with its widespread use, it has been found that the occurrence and incidence of adverse reactions after trientine treatment were comparable to those observed after D-penicillamine treatment, which requires further investigation. Potential adverse effects during trientine treatment include pancytopenia, hemorrhagic gastritis, loss of taste, systemic lupus erythematosus, and worsening of neurological symptoms. About 26% of patients with WD experience those corresponding symptoms during initial treatment [1, 2, 18].

### 12.3 Drugs that Reduce Copper Absorption

#### 12.3.1 Zinc Salts

Zinc salts inhibit the absorption of dietary copper by increasing the expression of metallothionein in enterocytes [1, 18]. Zinc acetate is approved for treating WD, while zinc sulfate is a dispersible formulation [16]. Given reports of progression of liver disease in patients treated with zinc monotherapy, the role of zinc salts in the treatment of WD remains controversial [81]. Marcelini *et al*. reported that zinc salts can effectively control WD and prevent its progression over a 10-year period, and reduce hepatic copper concentrations [82]. Nevertheless, Santiago *et al*. found elevated serum transaminase levels in 18/23 (78%) of children diagnosed through family screening [83]. The use of zinc gluconate in treating WD was first proposed by Prof. Yang RM [22] and, indeed, found rarely a few successful WD cases for decades years only with zinc.

Zinc induces metallothionein production in intestinal epithelial cells. Metallothioneins have a high affinity for copper and easily bind copper in intestinal intestinal epithelial cells. Metallothionein-bound copper cannot be absorbed and is excreted as intestinal epithelial cells are shed. Copper can enter the gastrointestinal tract with digestive fluids, where it cannot be reabsorbed; therefore, zinc can remove copper stored in the body and achieve a negative copper balance [1, 18]. Furthermore, absorbed zinc can also induce metallothionein production in hepatocytes, thus mitigating copper toxicity. The long-term efficacy of zinc in the treatment of WD is reliable, but it acts too slowly. Its effects can be achieved 1 to 3 months after treatment. The efficacy of different zinc supplements does not differ markedly, but their tolerability varies. Zinc acetate and zinc gluconate have less adverse effects [1, 2, 18].

##### 12.3.1.1 Indications

Zinc is used primarily as initial treatment in asymptomatic WD patients and maintenance therapy in symptomatic patients and pregnant patients, as well as patients with D-penicillamine intolerance. For the initial treatment of WD patients with severe acute presentation, zinc should not be used alone; instead, it should be used in combination or alternately with other chelating agents [2, 18, 22].

##### 12.3.1.2 Dosing and Administration

< 6 years of age: 25 mg twice daily.

6-16 years of age or < 50 kg of body weight: 25 mg three times daily

>16 years of age or >50 kg of body weight: 50 mg three times daily

The dose for adults and older children is 150-220 mg/d of elemental zinc [2, 18], equivalent to zinc gluconate tablets (70 mg/tablet, each tablet containing 10 mg of elemental zinc), administered orally in 3 divided doses. The dose for children aged 5-15 years with a body weight of < 50 kg is 75 mg/d of elemental zinc in 3 divided doses. For children aged < 5, the dose is 50 mg/d of elemental zinc in 2 divided doses. Since food may interfere with zinc absorption, it should be taken on an empty stomach. In case of gastric intolerance, zinc can be given at 0.5-1 h after a meal and gradually adapted to the administration mode on an empty stomach. If zinc is used in combination with a chelating agent, it should be administered at a time interval of 2 hours at least in order to avoid the neutralization of zinc efficiency by a chelating agent [2, 18, 22].

##### 12.3.1.3 Evaluation of Treatment Efficacy

The treatment efficacy of zinc is assessed according to patients’ clinical signs and symptoms, biochemical improvement, and changes in 24-h urinary copper excretion. If transaminase elevation occurs during zinc treatment, chelating agents such as D-penicillamine should be applied timely. Urinary copper decreases gradually after zinc treatment. The target range of 24-h urinary copper excretion on maintenance therapy is < 75 μg/24 h. Urinary zinc excretion can be measured to check treatment adherence to zinc treatment in WD patients. Urinary zinc excretion is typically 2,000-8,000 μg/24 h during treatment. Urinary copper excretion > 100 μg/24 h and urinary zinc excretion < 1,000 μg/24 h suggest poor adherence to zinc treatment [2, 18, 22].

##### 12.3.1.4 Adverse Effects

The adverse effects include nausea, abdominal pain, gastritis and gastric ulcers, and paradoxical worsening of neurological symptoms. Zinc has relatively few adverse effects, mainly including symptoms such as nausea and epigastric discomfort, some of which may affect patient's adherence. If a patient cannot tolerate zinc treatment, a low dose of zinc can be given initially and then slowly increased to the target dose [18, 22].

#### 12.3.2 Ammonium Tetrathiomolybdate (TTM)

TTM is a potent, fast-acting copper chelating agent. It can inhibit intestinal copper absorption, promote biliary copper excretion, and form a complex with copper and albumin in the blood, thus preventing the cellular uptake of copper and rapidly reducing serum-free levels. TTM has been evaluated in clinical trials, and preliminary results suggest that TTM rarely causes worsening of symptoms [1, 2, 18].

#### 12.3.3 Traditional Chinese Medicine

The team led by Prof. Yang RM, for the first time, used Gandou decoction to treat WD [22]. The combined use of Gandou decoction together and anti-copper therapy has better efficacy in the treatment of WD, especially with the ratio of high zinc *vs.* lower copper contents [22]. It has been shown that traditional Chinese medicine is beneficial in improving liver fibrosis and have some effects of hepatoprotection, as well as relieving constipation, so as to excrete mainly stool-copper in WD patients [22, 84]. A study reported that Gandou decoction combined with sodium dimercaptosulphonate can improve liver function and exert antifibrotic effects by inhibiting the serum tissue inhibitor of metalloproteinase-1 (TIMP-1) level, increasing the matrix metalloproteinase-1/TIMP-1 ratio in WD patients [84]. The mild urine anticopper effect of traditional Chinese medicine is generally considered an alternative or complementary treatment rather than a standalone or primary therapy [22].

### 12.4 Symptomatic Treatment of Internal Medicine

WD patients presenting with liver injury can be treated appropriately with hepatoprotective therapy. Patients with neuropsychiatric symptoms can be treated symptomatically under the guidance of neurologists. For example, dystonia and limb rigidity can be treated with amantadine, trihexyphenidyl, dopamine-based drugs, and baclofen; tremor can be treated with drugs such as trihexyphenidyl and dopamine-based drugs; chorea and athetosis can be treated with clonazepam and haloperidol; patients experiencing extreme excitement and mania can be given quetiapine, olanzapine, risperidone, and clozapine; patients with apathy and depression can be given antidepressant drugs. Patients who develop neuropsychiatric symptoms can also receive personalized rehabilitation treatment according to their conditions [18, 22].

### 12.5 Splenectomy

Prof. Yang RM, a pioneer in hepatolenticular degeneration research in China, for the first time, proposed the use of splenectomy to treat WD [85]. WD patients may develop hypersplenism. Surgical splenectomy is the conventional treatment for hypersplenism. A retrospective follow-up study of 70 WD patients presenting with hypersplenism who underwent splenectomy found that the platelet and white blood cell counts improved markedly in all patients after splenectomy, with no serious postoperative complications noted [85]. And quantitative analysis of 37 patients with neurological symptoms using the Unified Wilson's Disease Rating Scale revealed that patients’ neurological symptoms did not change in the short term (one week) after splenectomy. Obviously, it improved in the long term (one year) after splenectomy. Additionally, gastroesophageal varices improved significantly in the majority of patients after 1 year of splenectomy compared to before surgery [85]. A study involving 86 WD patients with hypersplenism (including 40 patients who underwent splenectomy and 46 patients who did not) showed that splenectomy markedly raised the platelet count, improved live function, and increased the survival rate of patients without neurological deterioration [86].

It is worth noting that portal venous system thrombosis occurs after splenectomy in WD patients and can lead to serious complications [87]. Furthermore, splenectomy may induce mood disorders and worsening of neurological symptoms. In recent years, partial splenic artery embolization has been increasingly used as a non-surgical alternative treatment for hypersplenism. A study revealed significant improvements in platelet and white blood cell counts in all patients after partial splenic embolization and splenectomy. Partial splenic embolization was associated with improved liver function without severe complications, mood changes, and neurologic deterioration. Conversely, seven patients with WD experienced worsening neurological symptoms after splenectomy [88].

### 12.6 Liver Transplantation

Liver transplantation is a life-saving treatment for WD that restores liver function and alleviates portal hypertension. Indications for liver transplantation in WD patients include ALF or end-stage liver disease that cannot be treated with medications [1, 2, 22]; on the other hand, liver transplantation may possibly be efficient in the treatment of the intractable neurological WD symptoms [18]. The New Wilson index based on AST, albumin, bilirubin, international normalized ratio, and white blood cell count is very accurate in predicting mortality, and patients with an NWI score of ≥11 need liver transplantation [89, 90]. During this period, in addition to intensive care, the optimal management of individuals with ALF includes aggressive fluid resuscitation, sepsis control, and continued reassessment of progressive organ dysfunction. Plasma exchange, haemofiltration, and blood exchange transfusion can provide protection against copper-mediated renal tubular injury and can be used as a bridge to liver transplantation [1, 2, 18]. Liver transplantation is indicated for approximately 5% of WD patients with ALF as the initial presentation, most commonly in the second decade of life, or those who develop end-stage liver disease and severe hepatic insufficiency, most commonly in the third and fourth decades of life. Liver transplantation restores normal biliary copper excretion, thus preventing disease recurrence [1, 2, 18]. It can also facilitate copper removal from extrahepatic sites. The outcomes of both cadaveric and living donor liver transplantation for WD are excellent [91]. Liver transplantation is also used to treat hepatocellular carcinoma in WD patients when tumor resection is not feasible. Liver transplantation solely for neurologic or psychiatric WD remains controversial. Living liver donation, cadaveric orthotopic, and auxiliary liver transplantation are options for transplantation in WD. WD patients can obtain excellent outcomes after liver transplantation, and supportive measures while awaiting transplantation contribute to a more successful outcome in patients [1, 18]. In the future, hepatocyte or stem cell transplantation, especially patient-derived induced pluripotent stem cells (iPSCs) /organoid managements, may augment or replace current liver transplantation for WD [92]. The Shanghai liver transplant team led by Academician Xia Qiang held the world's leading position in the field of pediatric liver transplantation, including numerous pediatric liver transplants that have been performed and a high survival rate after liver transplant in children Pediatric Liver Transplantation Global Census Group. 2023).

A healthy liver implanted by liver transplantation can provide WD patients with normal ATP7B protein, correct hepatic metabolic defects, and gradually reverse extrahepatic copper deposition, thus restoring normal liver function and alleviating portal hypertension. Liver transplantation can be considered in patients with ALF due to WD, as well as patients with decompensated cirrhosis who are not responsive or intolerant to anti-copper therapy [2, 18]. The short-term and long-term patients and graft survival after liver transplantation are high in both pediatric and adult WD patients. The 1-year and 5-year survival rates after liver transplantation were 90.1% and 89.0%, respectively, in pediatric WD patients, which were 88.3% and 86.0%, respectively, in adult WD patients, and there were no significant differences between the two patient groups [1, 2]. At present, there is still debate about the value or treatment efficacy of liver transplantation for WD patients presented with severe neurological symptoms (with or without cirrhosis) before surgery [2, 18]. There are few reports on the effects of living donor liver transplantation for WD, especially when the liver donor is a recipient’s relative carrying heterozygous ATP7B mutations, and limited data shows that its clinical effect is acceptable. Living donors (relatives of WD patients) are suggested to receive tests including serum ceruloplasmin, serum copper, and 24-h urinary copper excretion before transplantation [22]. Regular monitoring of serum copper and 24-h urinary copper is required in WD patients after liver transplantation to determine whether it is necessary to adhere to chelating agents, zinc, as well as a low-copper diet after surgery. It is recommended that WD patients who present with neurological symptoms before surgery continue to take a low-copper diet and receive low-dose zinc therapy after liver transplantation [1, 2].

### 12.7 Stereotactic Neurosurgery for Movement Disorders in WD

Stereotactic thalamotomy is considered a target treatment option for the control of extrapyramidal symptoms, such as severe tremors in WD patients. In China, Prof. Jianping Xu first used stereotactic thalamotomy to treat tremors in WD patients, and more than 10 cases have been treated [93]. In a case report, a 30-year-old WD patient with a severe postural and kinetic tremor in both hands was successfully treated with left stereotactic thalamotomy, improvement in tremor symptoms on both sides was observed [94]. The use of stereotactic thalamotomy for tremors in WD patients is limited to case reports [93]. Bilateral pallidotomy and bilateral globus pallidus deep brain stimulation may also help improve dystonia in patients with WD [94, 95].

The efficacy of this procedure in WD tremor treatment was illustrated by several case reports from the USA to Poland of Europe, as well as China, *etc*. However, further investigations should evaluate which group of patients will benefit most from those procedures. Importantly, patients should be informed that their neurological symptoms may mainly (nevertheless not all) be improving for several years on anti-copper treatment during the decision-making process for neurosurgical treatment [22, 95]. Severe, generalized dystonia, refractory to anti-copper treatment, and symptomatic pharmacological treatment may be treated with neurosurgical procedures, including DBS of globus pallidus internus (GPi), pallidotomy, or thalamotomy. Analogically, severe disabling Parkinsonian symptoms may be treated by DBS of GPi or subthalamic nucleus (STN) or the local lesions of these structures (Litwin *et al*. Tackling the neurological manifestations in Wilson's disease - currently available treatment options) [95].

### 12.8 Dietary Therapy

A low-copper diet has long been recognized as an important aspect of WD management [18, 22]. Nowadays, there are no randomized controlled clinical trials to support this strategy [18]. As a result, dietary management of WD patients conducted by clinicians varies widely in clinical practice. In a recent international survey by Sturm *et al*., the majority of respondents from North America suggest lifelong dietary copper restriction (<1 mg/day), whereas respondents from Europe suggest dietary copper restriction during the first year of treatment or until liver function tests return to normal (*Controversies and Variation in Diagnosing and Treating Children With Wilson Disease: Results of an International Survey. Journal of pediatric gastroenterology and nutrition 2016;63(1):82-7*). Nearly a quarter of the respondents from Europe do not advise patients to reduce dietary copper intake [2, 18]. A previous study indicates that eating chocolate, nuts, liver (and other offal), shellfish, and mushrooms should be avoided in WD patients [96]. WD patients in China, usually were advised to have relatively lower-copper diets whole-lifetime. Recently, Prof. Wang XP *et al*. published a review titled “Wilson's disease: food therapy out of trace elements” to discuss this issue.

### 12.9 Monitoring

Close monitoring of WD patients during pharmacotherapy is essential to ensure treatment efficacy and safety. In the first 3 months of treatment, treatment monitoring should be conducted 1-2 times a month in China hospital, this include assessing the symptoms and signs of the patients, changes in laboratory indicators (such as blood routine, urine routine, liver and kidney function, coagulation function, 24-h urinary copper, serum copper, ceruloplasmin, and serum-free copper, urinary zinc when zinc treatment is applied), enabling the assessment of treatment efficacy, adherence, and close observation of the adverse drug effects [22, 95]. In the Western countries, monitoring should be done every 1-3 months after symptom improvement and 2-3 times a year during the maintenance therapy [1, 17]. On maintenance therapy with chelating agents, the target range of 24-h urinary copper is 200-500 μg/24h, and serum-free copper is 100-150 μg/L, while on maintenance therapy with zinc, 24-h urinary zinc should be > 2 000 μg/24h, 24-h urinary copper should be < 75 μg/24h, and serum-free copper should be 100-150 μg/L [1, 2]. 24-h urinary copper excretion should be measured after 48 h of chelating therapy cessation if a 24-h urinary copper excretion of < 100 μg/24 h (indicating adequate anti copper effect) is achieved [1, 2, 22]. The K-F rings should be detected annually. Ultrasound of the liver (spleen) should be performed every 6 months [1, 22]. WD patients presenting predominantly with neurological manifestations should be evaluated using the unified Wilson disease rating scale and the modified Rankin Scale prior to treatment in order to assess the severity of neuropsychiatric symptoms [1, 18, 22]. MRI can be used to assess the severity of organic brain lesions, which can also used for disease monitoring to determine the degree of improvement or worsening of symptoms after treatment.

#### 12.9.1 Recommendations

1. Suitable anti-copper therapy should be initiated immediately upon diagnosis of WD, and the treatment should be lifelong (1A) [1, 22]. Initial treatment for symptomatic patients should include chelating agents (1A); chelating agents or zinc can be used for the treatment of asymptomatic patients or for maintenance therapy (1B).
2. Regular monitoring, including patients’ symptoms and signs, changes in laboratory indicators (such as blood routine, urine routine, liver and kidney function, coagulation function, 24-h urinary copper, serum copper, ceruloplasmin) should be performed 1-2 times per month during the first 3 months of treatment, every 1-3 months after symptom improvement, and 2-3 times a year during the maintenance therapy. The treatment regimens should be adjusted when abnormalities are noted (1B).
3. On maintenance therapy for WD, the target range of 24-h urinary copper is 200-500 μg/24 h with chelating agents (such as D-penicillamine), and < 75 μg/24 h with zinc.

## 13 MANAGEMENT OF SPECIAL POPULATIONS

### 13.1 Pregnant Patients

Female patients with WD require special consideration and counseling regarding conception, pregnancy, and breastfeeding. Preconception counseling should provide information to patients about the possibility of impaired fertility, the risk of spontaneous abortion, discussion of medication safety and compliance during pregnancy, and the need for genetic counseling [1, 2, 22]. Typically, mild anticoagulant therapy is considered safe in WD patients during pregnancy, but they have an increased risk of bleeding than the normal population. Pregnancy should be discouraged in WD patients with decompensated cirrhosis, as advanced liver disease poses a significant threat to the health of the mother and fetus [1, 18, 97]. Preparation for pregnancy in WD patients should include careful optimization of copper status. There have been concerns about the teratogenicity of chelation therapy, particularly penicillamine [97]. In China, congenital anomalies caused by maternal chelation therapy, including underdevelopment of internal organs and delayed wound healing, have been reported [22]. Conversely, discontinuation of chelation therapy during pregnancy may be associated with acute hepatic WD and ALF [95, 98]. Zinc therapy is relatively safe. The traditional Chinese medicine “Gandou decoction” contains *Rhubarb (Dahuang)*, which has the potential to induce miscarriage and should not be used in pregnant patients with WD, especially during early pregnancy [22]. Therefore, it is currently considered that the benefits of continuing chelation therapy throughout the entire pregnancy outweigh the theoretical risks. Currently, there is no definitive evidence that breastfeeding while undergoing chelation therapy is harmful or entirely safe. However, in mainland China, it is strictly prohibited. The use of chelation therapy in pregnant patients must be carefully considered in relation to potential risks and benefits.

WD patients may have an increased risk of miscarriage, but the majority of treated WD patients with stable clinical conditions can get pregnant. Suitable anti-copper therapy may be relatively safe for pregnant women with WD [1.2.18, 22]. Pregnant patients who adhere to anti-copper therapy have a lower rate of spontaneous miscarriage compared with untreated patients [1, 2]. Patients should be closely monitored for changes in disease condition changes and fetal development and routinely assessed for their risk of developing cirrhosis and portal hypertension. Given that the teratogenic risk of anti-copper therapy is highest during early pregnancy, partial-treatment interruption and reducing the dose of a metal chelating agent (by 25% to 50% of the pre-pregnancy dose) is recommended during this period [18, 22]. If treatment with zinc, pregnant patients with 24-h urinary copper excretion greater than 100 μg/24h should be administrated with 150 mg of elemental zinc per day orally in 3 divided doses; if the 24-h urinary copper is less than 100 μg, the dose can be reduced to 75 mg/d or even stop. There is currently no evidence that switching from a chelating agent to zinc prior to conception can reduce the risk of miscarriage or neonatal birth defects, and there is also a lack of supporting data, as systematic investigation in rare diseases is usually scarce [2, 18, 22]. Furthermore, some genetic advice should be raised if prenatal testing (including indirect biogenetic evidence from the mother's blood) reveals the presence of homozygous or compound heterozygous mutation in the ATP7B gene or other severe fetal defect [1, 2, 22]. Breastfeeding is not recommended for patients taking medications, as all available anti-copper drugs pass into the mother’s milk and may cause copper deficiency in the infant [22].

### 13.2 Asymptomatic Patients

For asymptomatic WD patients detected by family screening, a low-copper diet followed by early administration of zinc, traditional Chinese medicinal herbs, or DMSA with a low dose, and cautious use of anti-copper therapy with D-penicillamine is effective in preventing the onset of WD. If these patients have good long-term adherence to treatment, their survival rate is like that of the general population [2, 18, 22]. For asymptomatic infants and young children, zinc therapy can be initiated after 2 to 3 years of age. Zinc has traditionally been reserved for maintenance therapy of WD, but it is commonly used and now recommended by the AASLD as a first-line therapy for asymptomatic patients [1, 18]. Zinc can also be used as first-line and maintenance treatment for patients with prevalent neurological manifestations. Its slow action and mechanism of action are typically not associated with paradoxical worsening of neurological signs and symptoms [2, 18]. Zinc inhibits intestinal copper uptake by inducing the production of enterocyte metallothionein (an endogenous chelator of metals). Main copper is subsequently excreted into the feces, resulting in a negative copper balance [22]. Zinc also reduces hepatic copper toxicity by inducing the expression of metallothionein in hepatocytes, which may chelate copper in a nontoxic form or a form less prone to oxidative stress. With zinc therapy, urinary copper will be low, which is also an indicator of treatment adherence and treatment adequacy [1].

### 13.3 Patients with ALF

Truncating mutations in the ATP7B gene, female sex, and interruption of anti-copper drugs are associated with the development of ALF in WD patients. Although patients have markedly elevated serum bilirubin levels, they have normal or extremely low ALP concentrations and typically have hemolytic anemia, with normal 24-h urinary copper excretion [1, 2]. Liver transplantation is a life-saving treatment for WD patients with ALF, which can improve the long-term survival rate in such patients [22]. Indications for liver transplantation in patients with ALF due to WD are usually based on the modified New Wilson index. A score of ≥11 is associated with a high likelihood of death without liver transplantation. Plasma exchange, Molecular Adsorbent Recirculating System, and plasma transfusion combined with copper-chelating agents can also be used to treat patients with ALF due to WD, which can create conditions for successful transition to liver transplantation [22], even if patients have not fully recovered. It is worth noting that paradoxical neurological deterioration is sometimes observed in patients after liver transplantation. This may be associated with acute hemolysis and brain injury caused by a rapid increase in blood copper content during ALF [1, 2, 18]. Anesthetics used during liver transplantation can also cause neurological deterioration [95].

## 14 PROGNOSIS OF WD

Untreated WD patients can develop severe liver damage or neurological impairment, and the mortality rate of those patients was 5.0-6.1% higher than that of the general population. If WD can be diagnosed earlier, with long-term standardized copper restriction and anti-copper treatment being performed, the survival rate of asymptomatic WD patients (including those diagnosed by the screening for proband’s relatives) is similar to that of the general population [1, 2]; and the occurrence of complications can be delayed or even avoided in symptomatic WD patients, with obvious improvement in liver function and neurological symptoms, most WD patients can return to normal life and resume school or work. However, patients who do not follow the Doctor's Advice, take medications arbitrarily, or decide to stop treatment themselves will experience disease progression and may develop end-stage liver disease or severe neurological complications in a short period of time, even leading to death [18, 22].

## 15 FOLLOW UP

Treated WD patients should be followed up every 1-2 months in China, more often than in the Western countries [22, 95]. Patients with decompensated liver disease, severe neurological impairment, or non-adherence may be monitored more frequently. Follow up should include clinical assessment, body weight measurement, urine and blood tests (including complete blood count, liver function tests, coagulation profile, renal function, bone profile, ceruloplasmin, and serum copper). It may be helpful to video record neurological examinations in WD patients with movement disorders to monitor response to treatment [2, 18, 22]. Adherence and any broader concerns about medications should be addressed. 24-h urinary copper excretion should be measured annually or more frequently if there are concerns about clinical or biochemical deterioration. WD patients presented with K-F rings should be examined at the bedside and, if necessary, by an experienced ophthalmologist to confirm whether and when they can resolve [1]. Metabolic bone disease is common in WD, so vitamin D is encouraged to be supplemented [18, 22, 99, 100].

Some patients require high doses of chelating agents or combination therapy during the initial copper removal stage, but ideally, it should be changed to lower doses during maintenance therapy to reduce the risk of long-term overtreatment [18, 22]. Complications of chelation-induced iatrogenic copper deficiency, such as sideroblastic anemia and myelopathy, have been reported in overtreated patients. The dermatologic side effects of penicillamine, such as elastosis perforans serpiginosa or cutis laxa, are dose-dependent and further emphasize the need for careful review of maintenance doses of penicillamine. An ‘off treatment’ urinary copper excretion < 0.2 umol/24 h may indicate overtreatment [18]. Patients should be carefully monitored if the dose is reduced.

Some drugs commonly used to treat neurological and psychiatric symptoms, such as benzodiazepines, tricyclic antidepressants, and sodium valproate, are metabolized by the liver, which should be used with caution in patients with cirrhosis [1, 2]. Other medicines, such as antidepressants, can induce or exacerbate movement disorders [22]. Clinicians should also be aware that some neurological symptoms, particularly tremor [101], are more likely to improve with chelation therapy than other neurological symptoms [18, 22]. However, symptomatic treatments, either through beneficial or adverse effects, can make interpretation of the clinical response to chelation therapy more challenging. The use of deep brain stimulation for disabling tremors and dystonia has been reported in case reports, and the effect of deep brain stimulation in the treatment of WD has been evaluated in ongoing clinical trials (Registration No. NCT02552628). ATP7B gene therapy of autologous reprogrammed hepatocytes has been basically tried from mouse to human cells out of neuron/hepatocyte [102-106]. This invasive treatment may be appropriate for a small number of carefully selected patients who have reached a plateau in neurological recovery despite at least several years of intensive chelation therapy [18, 22]. Obviously, there are also differing opinions on this issue.

### 15.1 Recommendations

1. Pregnancy in WD patients with stable conditions is safe, and the majority of the patients can get pregnant (1A); WD patients who are effectively treated with suitable anti-copper therapy have a higher probability of successful pregnancy than untreated WD patients (1A); anti-copper therapy may be continued during pregnancy, the dose of chelating agents should be reduced, and the administration of traditional Chinese medicine *Gandou* tablet should be discontinued during the early pregnancy stage and 3 months before delivery (1A), and some patients can be treated only with zinc therapy.
2. For asymptomatic or pre-symptomatic WD patients, mild anti-copper therapy should be given early (1B).
3. Patients with ALF and decompensated cirrhosis who fail to respond or are intolerant to anti-copper treatment should undergo evaluation for liver transplantation (IA); WD patients with ALF are temporarily not allowed to receive liver transplantation. They should be treated initially with a combination of artificial liver therapy, such as plasma exchange and copper-chelating agents, before transitioning to liver transplantation (1B).

## 16 UNRESOLVED ISSUES

1. The factors influencing ATP7B variant penetrance and the underlying regulatory mechanisms need to be elucidated. Further investigation is needed to explore whether anti-copper therapy should be started immediately in patients who are diagnosed with WD through family screening for ATP7B gene mutation and do not have clear damage to major organs, such as the liver and nervous system. Follow-up and management programs for such patients need to be developed.
2. The diagnostic criteria for WD need to be further improved, and the Leipzig scoring system needs to be further validated and optimized in a large sample of patients.
3. Diagnostic problems in special populations with WD. For example, in rapid diagnosis of ALF, it is difficult to make a definite diagnosis in children, especially those younger than 5 years old, as their symptoms and signs, as well as biochemical test results, may be normal.
4. Development and validation of new diagnostic indicators for WD. The diagnostic value of indicators such as the novel neuroimaging methods, radioactive copper ratio, and relative exchangeable copper (exchangeable copper/total serum copper) for WD needs to be investigated and validated.
5. Large-sample, multi-center studies are needed to compare and investigate the efficacy of currently used chelating agents and zinc for WD and further clarify their indications. It is also necessary to explore whether the combined and sequential use of chelating agents and zinc are superior to single drug application and investigate the timing and specific methods of switching between different drugs.
6. The effect of the new treatment methods for WD, such as the new anti-copper drugs, cell transplantation or stem cell transplantation therapy or organoid management, and gene therapy, need to be investigated through global multi-center studies.
7. Issues in the management of patients after liver transplantation. Some cases’ findings from previous literature and our study suggested that new onset of neuropsychiatric symptoms indeed occurred after liver transplantation, as same as the strong anti-copper effects.

## CONCLUSION

WD is a rare disease that is treatable. It is estimated that 80,000-100,000 individuals with WD are in mainland China, and approximately 30,000 cases are suspected in the USA, as well as the UK. Recently, more than a thousand cases of the disease have been identified. For Anticopper drugs and other non-drug surgical Management, there are more choices in China, *e.g*., DMSA, DMPS, traditional Chinese medicines (Gandou decoction), and especially splenectomy & other operations. It is emphasized that there is much more experience for the extensive application of trientine in Western countries, and the trientine tetrahydrochloride has just been approved in mainland China. Ultimately, our goal is to provide the most suitable service up to date, for best WD friends of ourselves.

## Figures and Tables

**Fig. (1):**
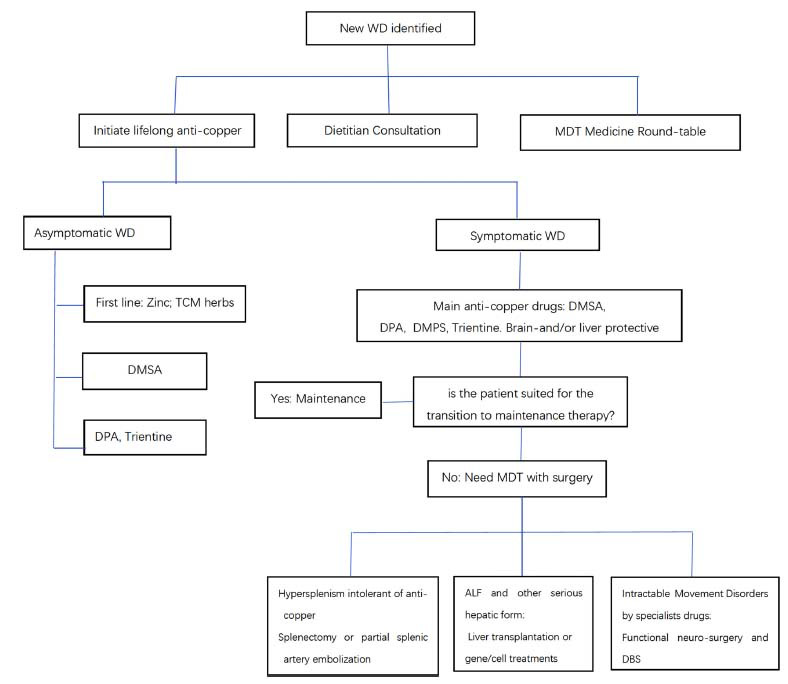
Map of Multiply-Department team for WD. **Abbreviations**: WD: Wilso’s disease, TCM: Traditional Chinese Medicine; DPA:D-Penicillamine. DMSA: dimercaptosuccinic acid; DMPS: Sodium dimercaptosulphonate; ALF; DBS: Deep Brain Stimulation.

**Table 1:** Application of the main anti-copper drugs.

**-**	**Dosing**	**Adverse Effects**
Penicillamine	Children: 125-250 mg/day, increased slowly by dose increments of 125-250 mg/week to an initial target dose of 20 mg/kg/day in two divided doses (maximum 1500 mg/day). Adults without neurologic or psychiatric symptoms: Initial target dose of 1000-1500 mg/day in two divided doses. Adults with neurological or psychiatric symptoms: 125-250 mg/day, increased slowly by dose increments of 125-250 mg/week to an initial target dose of 1000-1500 mg/day. Maintenance dose (typically after 2 years): 10-20 mg/kg/day in two divided doses.	Early reactions: allergic reactions (such as fever, rash), proteinuria, myelosuppression (neutropenia, thrombocytopenia), smell and taste disorders, paradoxical neurological worsening. Later reactions: lupus-like syndrome, Goodpasture’s syndrome, elastosis perforans serpiginosa, and poor wound/surgical incision healing.
Dimercaptosuccinic acid, DMSA	WD patients with varying degrees of liver injury, or neuropsychiatric symptoms, as well as those who are allergic or intolerant to D-penicillamine. It can be used in combination with zinc in different day-time or used alternately with D-penicillamine. The oral dose is 750-1,000 mg/d for adults and 10-20 mg/kg/d for children, administered orally, in 2 divided doses before meal. Foror long-term maintenance treatment.	Fewer adverse effects, mainly including mild gastrointestinal symptoms, such as nausea, vomiting, abdominal distension, loss of appetite, halitosis. Rash, itching, transient thrombocytopenia and transaminase elevation can occur in a few patients.
Sodium dimercaptosulphonate, DMPS	The adult dose is 500-750 mg, dissolved in 500 ml of 5% glucose injection and slowly administered Intravenously Pulse treatment joined in maintenance treatment, once a day for 5 consecutive days as a course of treatment; With a 2-d interval, multiple treatment courses can be repeated. The dosage for children is 10-20 mg/kg/day, beginning in 5 mg/kg/day.	Rapid intravenous infusion may result in symptoms such as nausea, tachycardia, and dizziness. They are alleviated by slowing down the infusion rate. Some allergic reactions such as rash, chills, fever mildly. Treatment should be paused during menstruation and gastrointestinal bleeding. and gastrointestinal bleeding.
Trientine (tetrahydrochloride)	Children: 150-200 mg/day, slowly increasing by 225-600 mg/day for trientine tetrahydrochloride (Cuprior) in two divided doses. Adults without neurological or psychiatric symptoms: 450-975 mg/day for trientine tetrahydrochloride (Cuprior) in two divided doses. Adults with neurological or psychiatric symptoms: 450-975 mg /day for trientine tetrahydrochloride (Cuprior) in two divided doses. Maintenance dose (typically after two years): 450-975 mg /day for trientine tetrahydrochloride (Cuprior).	Rticaria/rash, arthralgia/myalgia, proteinuria, haematuria, sideroblastic anaemia, and paradoxical worsening of neurological symptoms.
Traditional Chinese Medicinal Herbs (Gandou decoction/ tablets)	Only one kind of traditional Chinese medicine is beneficial to improve liver fibrosis in WD patients, besides its anti-copper effects similar to Zinc. It contains Rhubarb, Turmeric, *Houttuynia cordata*, *Poria cocos*, *Alisma orientalis*, *et al*.	Nausea, diarrhea, induced miscarriage in early pregnancy, especially by *Rheum officinale, M. officinale*.
Zinc salts	< 6 years of age: 25 mg twice daily. 6-16 years of age or < 50 kg of body weight: 25 mg three time daily. >16 years of age or >50 kg of body weight: 50 mg three times daily.	Nausea, abdominal pain, gastritis and gastric ulcers, and paradoxical worsening of neurological symptoms.

**Note**: The doses of penicillamine and zinc salts are based on experience, while the doses of trientine dihydrochloride and tetrahydrochloride are based on summary of product characteristics. The recommended dosage of trientine dihydrochloride and tetrahydrochloride is expressed in mg of trientine base, not in mg of trientine salt.

## LIST OF ABBREVIATIONS

CPM: Central Pontine Myelinolysis
DMSA: Dimercaptosuccinic Acid
GPi: Globus Pallidus Internus
INR: International Normalized Ratio
iPSCs: Induced Pluripotent Stem Cells
LSM: Liver Stiffness Measurement
NAFLD: Non-alcoholic Fatty Liver Disease
STN: Subthalamic Nucleus
TB: Total Bilirubin
TIMP-1: Tissue Inhibitor of Metalloproteinase-1
TTM: Tetrathiomolybdate
WD: Wilson's Disease
